# Differential Cloud Particles Evolution Algorithm Based on Data-Driven Mechanism for Applications of ANN

**DOI:** 10.1155/2017/8469103

**Published:** 2017-07-06

**Authors:** Wei Li

**Affiliations:** School of Computer Science and Engineering, Xi'an University of Technology, Xi'an 710048, China

## Abstract

Computational scientists have designed many useful algorithms by exploring a biological process or imitating natural evolution. These algorithms can be used to solve engineering optimization problems. Inspired by the change of matter state, we proposed a novel optimization algorithm called differential cloud particles evolution algorithm based on data-driven mechanism (CPDD). In the proposed algorithm, the optimization process is divided into two stages, namely, fluid stage and solid stage. The algorithm carries out the strategy of integrating global exploration with local exploitation in fluid stage. Furthermore, local exploitation is carried out mainly in solid stage. The quality of the solution and the efficiency of the search are influenced greatly by the control parameters. Therefore, the data-driven mechanism is designed for obtaining better control parameters to ensure good performance on numerical benchmark problems. In order to verify the effectiveness of CPDD, numerical experiments are carried out on all the CEC2014 contest benchmark functions. Finally, two application problems of artificial neural network are examined. The experimental results show that CPDD is competitive with respect to other eight state-of-the-art intelligent optimization algorithms.

## 1. Introduction

Optimization problems in engineering are often very complex and difficult to solve. At first, lots of deterministic algorithms based on mathematical programming theory are used for engineering optimization problems. They obtain better results for relative simple and ideal models. Unfortunately, these deterministic algorithms show poor performance for real-world complex problems. Inspired by natural evolution, more and more researchers are interested in the development of nature-inspired algorithm by exploring natural phenomena [1]. These natural phenomena mainly include the biological evolutionary process, animal behavior, and physical phenomena. The nature-inspired algorithms can solve the difficult design and optimization problems by building solutions that are more fit relative to desired properties [2]. The resulting field, nature-inspired algorithms have been successful in solving optimization, design, constrained, large-scale, and multiobjective clustering and forecasting [3–9].

Evolutionary algorithms (EAs), such as Genetic Algorithm (GA) [10], Differential Evolution (DE) [11], and Derandomized Evolution Strategy with Covariance Matrix Adaptation (CMA-ES) [12], are inspired by the biological evolutionary process. GA, proposed by Fraser and popularized by Holland, has been widely studied. It solves optimization problems by simulating Darwinian evolution concepts, such as crossover, mutation, and selection. DE, proposed by Storn and Price, is a simple yet powerful population-based evolutionary algorithm. CMA-ES, proposed by Hansen and Ostermeier, adapts the complete covariance matrix of the normal mutation distribution. Swarm intelligence (SI) algorithms, such as Particle Swarm Optimization (PSO) [13, 14], Artificial Bee Colony (ABC) [15], Teaching-Learning-Based Optimization (TLBO) [16, 17], and Jaya (a Sanskrit word meaning victory) algorithm [18], are inspired by all kinds of animal behavior. PSO explores the search space according to* pbest* and* gbest*, which are the past best position and the global best position achieved by particles, respectively. ABC, proposed by Karaboga, simulates the foraging behavior of the honeybee swarm and has been applied to solve many engineering optimization problems [19, 20]. The TLBO method, proposed by Rao, is based on the effect of the influence of a teacher on the output of learners in a class [16]. In order to reduce the complexity of the algorithm, Rao [18] proposed Jaya algorithm which uses one phase instead of two phases of the TLBO algorithm. Jaya algorithm tries to get closer to reaching the best solution and tries to move away from the worst solution. Other heuristic methods, such as biogeography-based optimization (BBO) [21], Simulated Annealing (SA) [22, 23], Chemical Reaction Optimization (CRO) [24], and Brain Storm Optimization (BSO) [25], simulate the physical phenomena or rules. BBO, proposed by Simon in 2008, is a newly proposed metaheuristic algorithm. In BBO, mathematical models are used to describe the evolution process of species, such as migration, mutation, and distribution of species. A solution is regarded as an island (habitat) with a habitat suitability index (HSI). Islands with a high HIS are well suited for species, and vice versa. Suitability index variables (SIV), which refer to the features correlated with HSI, and HSI are considered as the search space and objective function, respectively [26]. SA is a heuristic algorithm which is based on an analog of thermodynamics with the way metals cool and anneal [27]. CRO is a chemical-reaction-inspired metaheuristic which mimics the characteristics of chemical reactions in solving optimization problems [24]. BSO mimics the brainstorming process in which a group of people solves a problem together [28].

The issue of exploration-exploitation is the major factor which influences the performance of evolutionary algorithm. Exploration helps to find new potential solutions and improve the convergence rate of algorithm. Exploitation helps to improve the quality of found-so-far solutions. However, overexploration may lead to slow convergence and overexploitation may increase the risk of premature convergence. Therefore, numerous ideas are proposed to balance the exploitation and exploration search process of EAs [29]. For example, a large scaling factor *F* in DE is required at the early stage of the evolution to ensure strong exploration capability, while a small *F* is preferred to improve exploitation capability at the later stage [30]. Therefore, many improved DE variants, such as adaptive differential evolution with optional external archive (JADE) [31], self-adaptive control parameters in differential evolution (jDE) [32], a self-adaptive DE (SaDE) algorithm [33], and a composite DE (CoDE) algorithm [34], are proposed to improve the relationship of exploration and exploitation with the proper settings of control parameters [35–39]. For PSO, many different modified PSO variants, including inertia weight and the acceleration coefficient, are proposed by the researchers to enhance PSO's exploration capability and alleviate premature convergence problem [40–46].

Designing suitable evolutionary strategies and control parameters is important to realize a good balance between exploration and exploitation. In this paper, we proposed a novel nature-inspired algorithm called differential cloud particles evolution algorithm based on data-driven (CPDD) mechanism for solving global optimization problems. The CPDD algorithm simulates the change of matter state and cloud transformation process. The optimization process is divided into two stages, namely, fluid stage and solid stage. The CPDD algorithm carries out the strategy of integrating global exploration with local exploitation in fluid stage. Furthermore, local exploitation is carried out mainly in solid stage. Data-driven mechanism is designed for obtaining better control parameters to keep a better balance between exploration and exploitation.

The rest of the paper is organized as follows.  Section 2 introduces the proposed CPDD and the concepts behind it in detail. In Section 3, the performance of the proposed CPDD is validated on different optimization problems.  Section 4 shows the applications for training artificial neural network. Finally, the conclusions and ideas for future research are drawn up in Section 5.

## 2. Differential Cloud Particles Evolution Algorithm Based on Data-Driven Mechanism

### 2.1. Algorithm Background

The idea of differential cloud particles evolution algorithm based on data-driven mechanism is inspired from the phenomenon of matter state transition and cloud formation. For further understanding, an explanation on the principles of matter state transition and cloud formation will be stated as follows.

**Figure 1:**
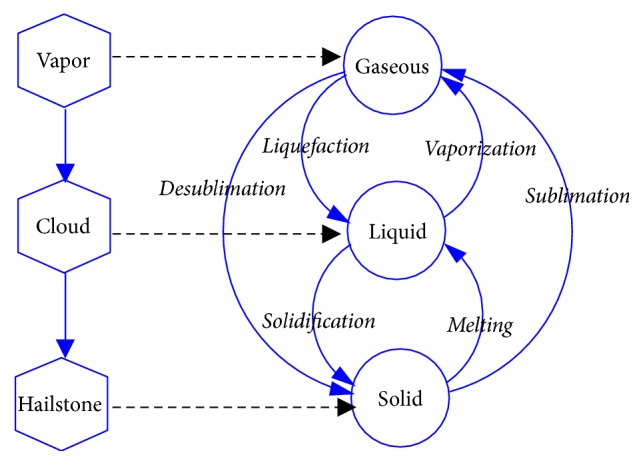
Cloud formation and matter state transition.

Substances commonly exist in three states: gaseous, liquid, and solid. For example, heat evaporates water into steam, while low temperature turns water to ice. Similarly, three states are involved in the formation and change of cloud. The water evaporated into vapor. Water vapor condenses to form clouds. The clouds transform into snow or hailstone with decreasing temperature. Therefore, water vapor, cloud, and snow can be regarded as gaseous, liquid, and solid, respectively. The cloud transformation process and matter state transition are illustrated in Figure 1. As a result, the matter shows different states with different temperature.

CPDD loosely simulates the cloud transformation process and matter state transition. Because the gas and the liquid have fluidity, there are two types of operation implemented in CPDD, namely, fluid stage operation and solid stage operation. We utilize the term “phase transition” to describe transitions between fluid stage and solid stage. A phase transition is the transformation of a thermodynamic system from one phase or state of matter to another one by heat transfer.

### 2.2. The Proposed CPDD

Similar to other metaheuristic algorithms, the proposed algorithm begins with an initial population called the cloud particles. Each cloud particle in CPDD algorithm represents a solution in the population. The population size is similar to the temperature in real world. Liquefaction refers to the process in which gas is transformed into liquid. Solidification is a phase transition in which liquid is turned into solid. Liquefaction and solidification need to give off heat. The temperature will gradually decrease in the exothermic process. In order to improve the performance of the algorithm, EAs had better to start with exploration and then gradually change into exploitation [47]. Therefore, CPDD algorithm has a larger population to ensure strong exploration ability at the beginning of the evolution. The population size gradually decreased and the exploitation ability gradually improved during evolution. In the later evolution, CPDD algorithm has a smaller population to encourage broader exploitation. The change of population number is similar to the change of the temperature; namely, matter has high temperature in gas state and has low temperature in solid state.

#### 2.2.1. Fluid Stage

**Figure 2:**
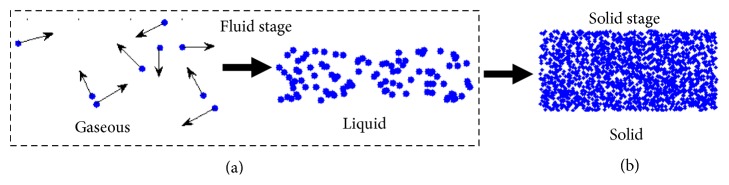
Three states of cloud particles.

The fluid stage, which mimics the movement of the fluid, includes gaseous state and liquid state. At the beginning, the initial state is gaseous state, which is composed of many cloud particles. Each cloud particle has good fluidity and can move freely. Therefore, the algorithm shows strong exploration ability in gaseous state. For the evolutionary process, the population size gradually decreases. Then, the local exploitation ability can be improved by reducing the population size. In addition, the state of population transforms from gaseous state into liquid one as the cloud particles move. The movement of cloud particles in liquid state causes macrolocal exploitation. The movement of cloud particles in fluid stage is given in Figure 2(a).

Inspired by JADE, an improved search strategy is introduced in fluid stage. Similar to other evolutionary algorithms for optimization problems, a CPDD population is represented as a set of real parameter vectors which is defined as follows:

(1) $$\begin{array}{c} x_{i}=\left(\;x_{1},x_{2},\ldots ,x_{D}\;\right),\;i=1,\ldots ,N, \end{array}$$

where *D* is the dimensionality of the optimization problem and *N* is the population size. In fluid stage, the search strategy, based on DE/current-to-*p*best with optional archive, is generated in the following manner:

(2) $$\begin{array}{c} v_{i}=x_{r1}+F_{i}\cdot \left(\;x_{best}-x_{r1}+x_{r2}-x_{r3}\;\right), \end{array}$$

where *i* ∈ {1,…, *N*}, *r*_1_, *r*_2_, and *r*_3_ are mutually different random integer indices selected from *i* ∈ {1,…, *N*}, and **x**_*best*_ is randomly chosen as one of the top 100*p*% cloud particles in the current population. *p* is 15% of the population size. **x**_*r*3_ is selected from the union of the population and the archive. The archive is the set of archived inferior solutions and is used for maintaining diversity in JADE [34]. If the archive size exceeds 150% of the population size, some solutions are randomly removed from the archive so that some newly cloud particles can be inserted into the archive. *F*_*i*_, which is different from *F* in JADE, is a mutation factor that controls the speed of algorithm process and is generated at each generation by data-driven mechanism introduced later in (4).

#### 2.2.2. Solid Stage

The algorithm has found the potential area of optimal solution after global exploration and macrolocal exploitation in fluid stage. Then the phase transition is carried out; namely, the population transforms from fluid into solid. The cloud particles vibrate only in a small region and carry out microlocal exploitation. The process, in which most of the cloud particles are gathered toward the optimal solution (microlocal exploitation), is analogous to the solidification. Finally, most of the cloud particles will gather to a position, that is, the location of the optimal solution. Figure 2(b) shows the movement of cloud particles in solid stage. The following strategy is search strategy used in solid stage:

(3) $$\begin{array}{c} v_{i}=x_{best}+F_{i}\cdot \left(\;x_{r2}-x_{r1}\;\right). \end{array}$$

Similar to fluid stage, **x**_*best*_ is randomly chosen as one of the top 100*p*% cloud particles in the current population. **x**_*r*2_ is selected from the union of the population and the archive. *r*_1_ and *r*_2_ are mutually different random integer indices selected from *i* ∈ {1,…, *N*}. *F*_*i*_ is generated at each generation by data-driven mechanism.

The population size is reducing from *N* to four in order to balance the exploration and exploitation. In fluid stage, the algorithm carries out global exploration and macrolocal exploitation. In solid stage, the algorithm performs macrolocal exploitation. Consequently, the algorithm can balance the exploration and exploitation based on the transformation from fluid stage to solid stage.

### 2.3. Control Parameters Assignments

In CPDD algorithm, the parameter *F* controls the diversity of the population. Higher value of the parameter *F* will increase the diversity of the cloud particles and enhance the convergence speed. On the contrary, smaller value of the parameter *F* will result in premature convergence and slow convergence rate. In addition, for different problems, different values of parameters are needed in different evolution stage. Based on our previous research [48], in this paper, *F* is produced based on data-driven mechanism which can be described as follows:

(4) f1=sin⁡ef02f2=r1×sin⁡1−f0⁡max⁡FES+sin⁡1−f0⁡Fi=r2×f2+f1.

Initial *F*_*i*_ is generated according to (4). *f*_0_ is a parameter and is set to 0.068. In each generation, *F*_*i*_ value that succeeds in generating a trial **v**_*i*_ which is better than the parent individual **x**_*i*_ is preserved and is recorded as *F*_*G*_. The size of *F*_*G*_ is recorded as |*F*_*G*_|. If |*F*_*G*_| exceeds the current population size, randomly selected elements are deleted. If |*F*_*G*_| is less than the current population size *N*, the (*N*- | *F*_*G*_|) new *F*′ is generated according to (4). Consequently, *F*_*i*_ value that shows better performance is preserved for next generation. It means that *F*_*i*_ value from last generation drives the evolution of new generation.

CR, the crossover rate, is another important control parameter in CPDD algorithm. The initial CR is a Gaussian distribution with mean “0.5” and standard deviation “0.1.” Similar to the parameter *F*, CR values that have performed well in generation *G* are preserved for next generation. The preserved CR is recorded as CR_*G*_. The size of CR_*G*_ is recorded as |CR_*G*_|. If |CR_*G*_ | = 0, CR_*i*_ is regenerated as follows:

(5) $$\begin{array}{c} {CR}_{i}=0.9+rand. \end{array}$$

In addition, the cloud particles transfer from fluid stage to solid stage when CR_G_ is empty.

If |CR_*G*_| is less than the current population size *N*, the (*N*- | CR_*G*_|) new CR′ is given by

(6) $$\begin{array}{c} {CR}^{\prime }=\sigma \left(\;{CR}_{G}\;\right)+rand, \end{array}$$

where *σ*(CR_*G*_) refers to the standard deviation of CR_*G*_. rand denotes a uniformly selected random number from [0,1). If |CR_*G*_ | > *N*, extra elements are randomly selected and deleted. Therefore, the next generation of CR is produced by CR_*G*_, which shows better performance in the last generation.

The pseudocode of CPDD is illustrated in Algorithm 1. Φ(0.5,0.1) refers to a Gaussian distribution with mean “0.5” and standard deviation “0.1.” Popsize is the current population size. FES stands for the number of function evaluations. maxFES stands for the maximum number of function evaluations.

## 3. Experiments and Discussions

### 3.1. General Experimental Setting

#### 3.1.1. Test Problems and Dimension Setting

For a comprehensive evaluation of CPDD, all the* CEC2014 Special Session on Real-Parameter Single Objective Optimization* [49] benchmark problems are used to test the performance of CPDD. The CEC2014 benchmark set consists of 30 test problems. According to their shape characteristics, these benchmark problems can be broadly classified into four kinds of optimization problems [49]:

- Unimodal problems *f*_1_–*f*_3_
- Simple multimodal problems *f*_4_–*f*_16_
- Hybrid problems *f*_17_–*f*_22_
- Composite problems *f*_23_–*f*_30_

For all of the problems, the search space is [−100,100]^D^. In this paper, the dimension (*D*) of all problems is set to 10 and 30.

#### 3.1.2. Experimental Platform and Termination Criterion

For all experiments, 30 independent runs are carried out on the same machine with a Celoron 3.40 GHz CPU, 4 GB memory, and windows 7 operating system with Matlab R2009b and conducted with *D* × 10,000 (number of function evaluations, FES).

#### 3.1.3. Performance Metrics

In our experimental studies, the mean value (*F*_mean_), standard deviation (SD), maximum value (Max), and minimum value (Min) of the* solution error measure* [50] which is defined as *f*(*x*) − *f*(*x*^*∗*^) are recorded for evaluating the performance of each algorithm, where *f*(*x*) is the best fitness value found by an algorithm in a run and *f*(*x*^*∗*^) is the real global optimization value of tested problem. In order to statistically compare the proposed algorithm with its peers, the statistical tool *t*-test [16] at a 0.05 significant level is used to evaluate whether the median fitness values of two sets of obtained results are statistically different from each other. Three marks “+,” “−,” and* “≈”* are also used to report the results clearly.“+,” “−,” and* “≈”* denote that the performance of CPDD is better than, worse than, and similar to that of the corresponding algorithm, respectively.

### 3.2. Comparison with Eight State-of-the-Art Optimization Algorithms on 10 and 30 Dimensions

In this part, CPDD is compared with PSO, PSOcf (PSO with constriction factor) [44], TLBO, DE, JADE, CMA-ES, ABC, and BBO. The appropriate parameters are important for the performance of the optimization algorithms. Therefore, the setting of parameters of different algorithms is given in the following.

For CPDD, *F* and CR are produced according to the data-driven mechanism. Initial *F* is produced according to (4). The initial CR is a Gaussian distribution with mean “0.5” and standard deviation “0.1.” The population size *N* is set to 13 × *D*. The maximum size of the archive is set to 1.5 × *N*. For DE, the population size *N* is set to 100. *F* and CR are set to 0.5 and 0.9, respectively. For PSO, the population size *N* is set to 40, the linearly decreasing inertia *ω* from 0.9 to 0.4 is adopted over the course of the search, and the acceleration coefficients *c*_1_ and *c*_2_ are both set to 1.49445. For ABC, the number of colony sizes is set to 20, and the number of food sources is set to half of the colony sizes. For JADE, the population size *N* is set to 100; *p* = 0.05 and *c* = 0.1. The parameters of other algorithms are the same as those used in the corresponding references.

The statistical results, in terms of *F*_mean_, SD, Max, and Min obtained in 30 independent runs by each algorithm, are reported in Tables 1~7.

#### 3.2.1. Unimodal Problems *f*_1_–*f*_3_

From the statistical results of Table 1, we can see that CMA-ES and CPDD achieve the optimal solution in each run for unimodal problems *f*_1_–*f*_3_ for 10 dimensions. CPDD performs better than other algorithms on *f*_2_-*f*_3_ and achieves the second best performance on *f*_1_ for 30. The reason that CPDD has the outstanding performance may be because of data-driven mechanism, which is helpful for obtaining better control parameters.

#### 3.2.2. Simple Multimodal Problems *f*_4_–*f*_16_

From Table 2, we observe from the statistical results that CPDD is significantly better than other algorithms on *f*_5_–*f*_7_, *f*_9_, *f*_13_, *f*_14_, and *f*_16_. ABC performs well on *f*_4_; JADE performs well on *f*_10_-*f*_11_ and *f*_15_, respectively. PSOcf performs well on *f*_12_. CPDD and JADE achieve the optimal solution on *f*_8_.  Table 3 shows that CPDD obtains better solutions than other algorithms on *f*_4_, *f*_6_, *f*_12_, and *f*_13_. CMA-ES performs well on *f*_5_. JADE performs well on *f*_8_–*f*_11_, *f*_15_, and *f*_16_. ABC performs well on *f*_14_. DE, JADE, and CPDD perform well and achieve the similar solutions on *f*_7_. 

#### 3.2.3. Hybrid Problems *f*_17_–*f*_22_

In the case of *f*_17_–*f*_22_, we find that CPDD achieves very competitive results from Tables 4 and 5. It beats PSO, PSOcf, TLBO, DE, JADE, CMA-ES, ABC, and BBO on these hybrid problems for 10 and 30 dimensions except *f*_17_. DE performs well on *f*_17_ for 10 dimensions. This may be due to the fact that the scheme of matter state change can help CPDD to keep a better balance between exploration and exploitation. 

#### 3.2.4. Composite Problems *f*_23_–*f*_30_

From Tables 6 and 7, we find that these composite problems are very time-consuming for fitness evaluation compared to others because these problems combine multiple test problems into a complex landscape. Therefore, it is extremely difficult for state-of-the-art optimization algorithms to obtain relatively ideal results.  Table 6 shows that CPDD obtains the better solutions on *f*_24_, *f*_25_, and *f*_27_ for 10 dimensions. ABC performs well on *f*_23_ and *f*_28_. For *f*_26_, all the algorithms can find the similar solutions except CMA-ES. ABC performs well on *f*_23_ and *f*_28_. DE performs well on *f*_29_. DE and CPDE achieve the similar solutions on *f*_30_. It can be concluded from Table 7 that all the algorithms can find the similar solutions on *f*_23_ except BBO. All the algorithms can find the similar solutions on *f*_26_ except PSO, PSOcf, and CMA-ES. CPDD obtains the better solutions on *f*_25_ and *f*_27_. DE obtains the better solutions on *f*_29_ and *f*_30_. TLBO performs well on *f*_24_.

**Figure 3:**
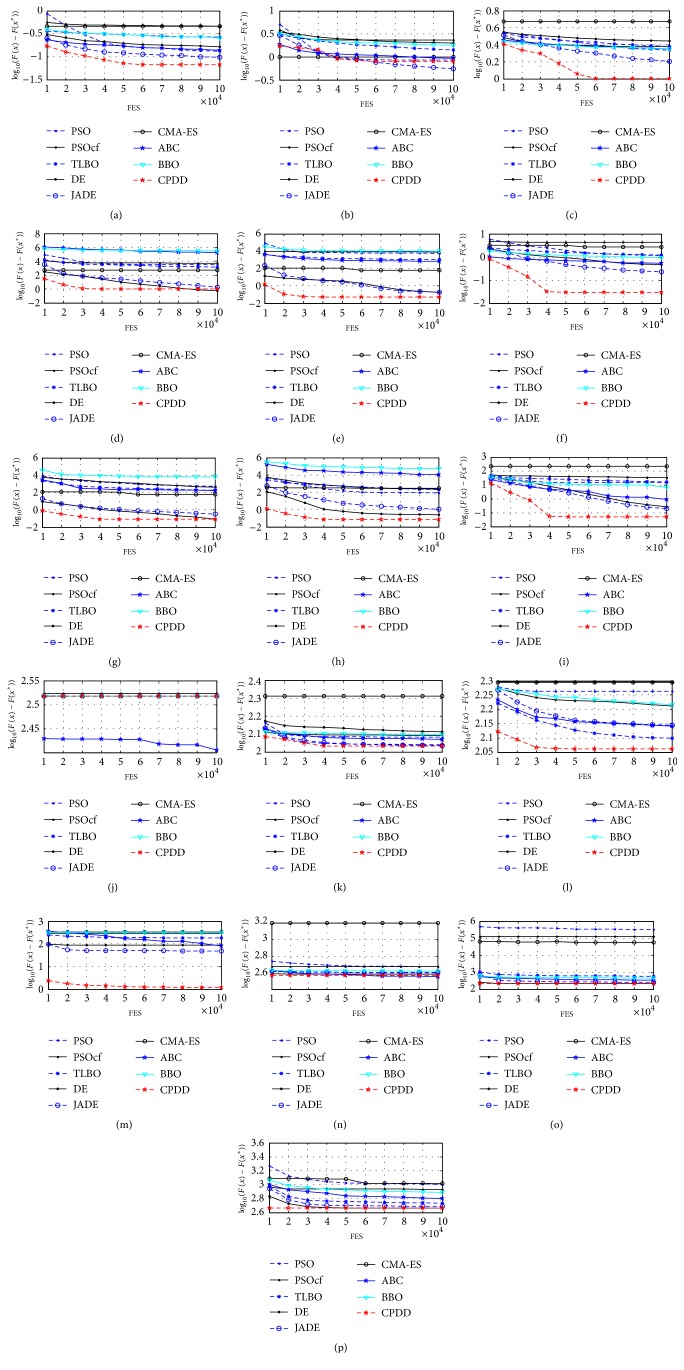
Evolution of the mean function error values derived from PSO, PSOcf, TLBO, DE, JADE, CMA-ES, ABC, BBO, and CPDD versus the number of FES on sixteen test problems of ten variables. (a) *f*_14_. (b) *f*_15_. (c) *f*_16_. (d) *f*_17_. (e) *f*_18_. (f) *f*_19_. (g) *f*_20_. (h) *f*_21_. (i) *f*_22_. (j) *f*_23_. (k) *f*_24_. (l) *f*_25_. (m) *f*_27_. (n) *f*_28_. (o) *f*_29_. (p) *f*_30_.

**Figure 4:**
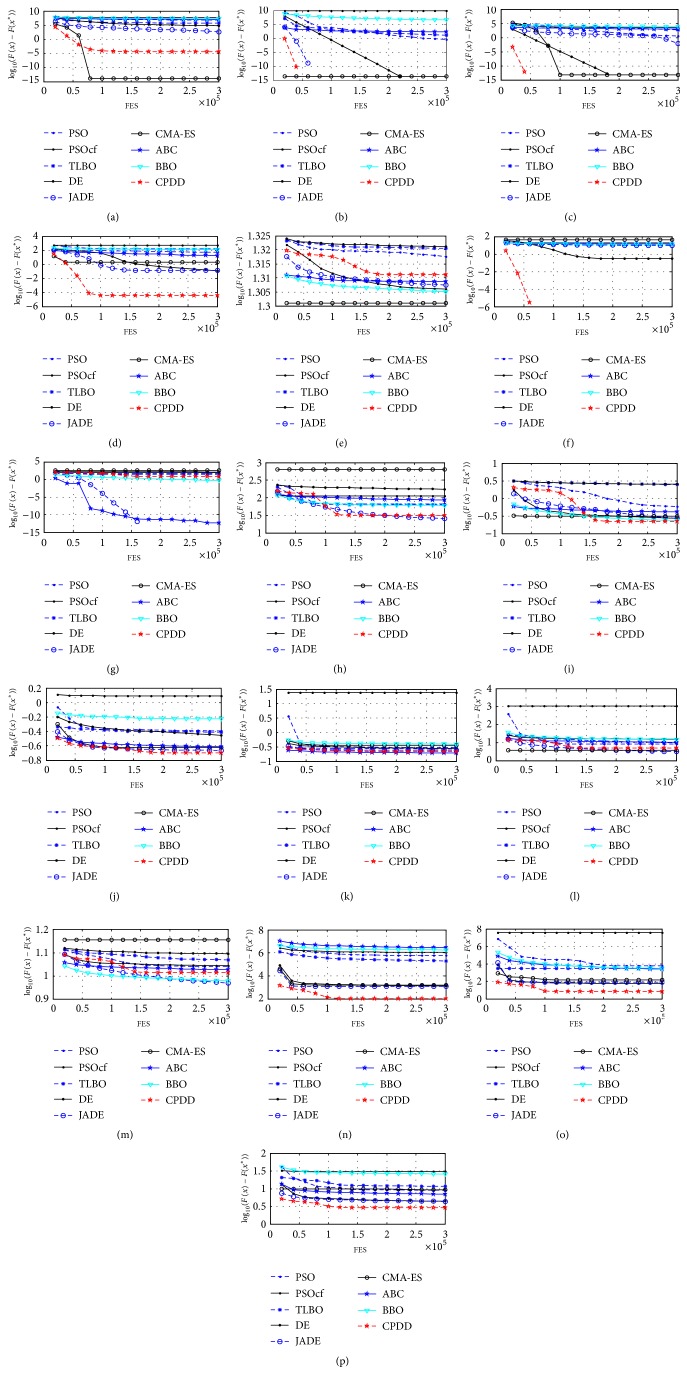
Evolution of the mean function error values derived from PSO, PSOcf, TLBO, DE, JADE, CMA-ES, ABC, BBO, and CPDD versus the number of FES on sixteen test problems of thirty variables. (a) *f*_1_. (b) *f*_2_. (c) *f*_3_. (d) *f*_4_. (e) *f*_5_. (f) *f*_6_. (g) *f*_8_. (h) *f*_9_. (i) *f*_12_. (j) *f*_13_. (k) *f*_14_. (l) *f*_15_. (m) *f*_16_. (n) *f*_17_. (o) *f*_18_. (p) *f*_19_.

According to the experimental results on thirty test problems from Table 8, we find that CPDD outperforms PSO, PSOcf, TLBO, DE, JADE, CMA-ES, ABC, and BBO on twenty-six, twenty-six, twenty-seven, twenty, eighteen, twenty-five, twenty-three, and twenty-five test problems, respectively, for 10 dimensions. CPDD outperforms PSO, PSOcf, TLBO, DE, JADE, CMA-ES, ABC, and BBO on twenty-eight, twenty-six, twenty-six, twenty, fourteen, twenty-two, twenty-one, and twenty-two test problems, respectively, for 30 dimensions. Moreover, Figures 3 and 4 have further displayed the convergence graphs of different benchmark problems in terms of the mean errors (in logarithmic scale) achieved by each of nine algorithms for CEC2014 problems versus the number of FES for 10 and 30 dimensions.

In summary, it is suggested that CPDD beats PSO, PSOcf, TLBO, DE, JADE, CMA-ES, ABC, and BBO in 15 out of 30 benchmark problems for 10 dimensions. CPDD achieves better performance than other seven algorithms in 14 out of 30 benchmark problems for 30 dimensions. The experiment results reveal that CPDD works well for most benchmark problems. This is due to the data-driven mechanism and the phase transition of matter mechanism which are used in CPDD. The data-driven mechanism makes use of the better control parameters which are found by the last generation to produce new control parameters for next generation. The experiment results indicate that the control parameters which are achieved by the data-driven mechanism are appropriate for most benchmark problems and are helpful for finding better solutions. The cloud particles carry out phase transition according to the extent of evolution. The exploration ability and exploitation ability of the algorithm are dynamically adjusted by the phase transition mechanism. Therefore, it not only can improve the convergence rate of algorithm but also can decrease the risk of premature convergence as much as possible.

## 4. The Real-World Optimization Problem

**Figure 5:**
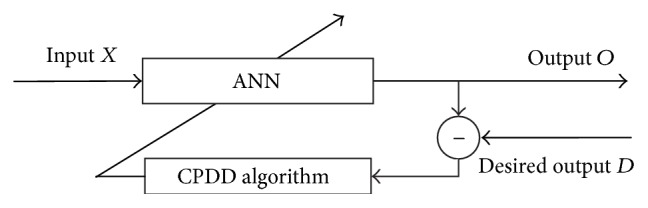
CPDD-based ANN.

In this section, the proposed CPDD algorithm is applied to estimate parameters of a real-world problem. The artificial neural network trained by our CPDD algorithm is a three-layer feed-forward network which includes input units, hidden units, and output units. The basic structure of the proposed scheme is depicted in Figure 5.

In the three-layer feed-forward network, input *X* = (*x*_1_, *x*_2_,…,*x*_*i*_,…,*x*_*n*_)^*T*^, output *O* = (*o*_1_, *o*_2_,…,*o*_*k*_,…,*o*_*t*_)^*T*^, and desired output *D* = (*d*_1_, *d*_2_,…,*d*_*k*_,…,*d*_*t*_)^*T*^. Comparison needs to be made to check out the difference between the test output and real demand. The aim of neural network training is to find a set of weights with the smallest error measure. The objective function is the mean sum of squared errors (MSE) over all training patterns which is shown as follows:

(7) $$\begin{array}{c} MSE=\frac{1}{Q*K}\overset{Q}{\underset{i=1}{\sum }}\overset{K}{\underset{j}{\sum }}\frac{1}{2}{\left(\;d_{ij}-o_{ij}\;\right)}^{2}, \end{array}$$

where *Q* is the number of training data sets, *K* is the number of output units, *d*_*ij*_ is desired output, and *o*_*ij*_ is output inferred from neural network.

### 4.1. SISO Nonlinear Function Approximation

In this example, there are one input unit, five hidden units, and one output unit in the three-layer feed-forward ANN. The model is constructed to model the curve of a nonlinear function which is described by the following equation [21]:

(8) $$\begin{array}{c} y=\sin\left(\;2x\;\right)\exp\left(\;-2x\;\right). \end{array}$$

The sigma function is used as activation function in the output layer. The number (dimension) of the variables is 16 for CPDD-based ANN. In order to train the ANN, 200 pairs of data are chosen from the real model. The population size is set as 50; the maximal number of function evaluations (FES = 10000) is used as ended condition of each algorithm. To assess the performance of each algorithm in noise, 30 db additive with Gaussian noise is added to the experiment. For each algorithm, 50 runs are performed. The other parameters are same as those of the previous investigations.

**Figure 6:**
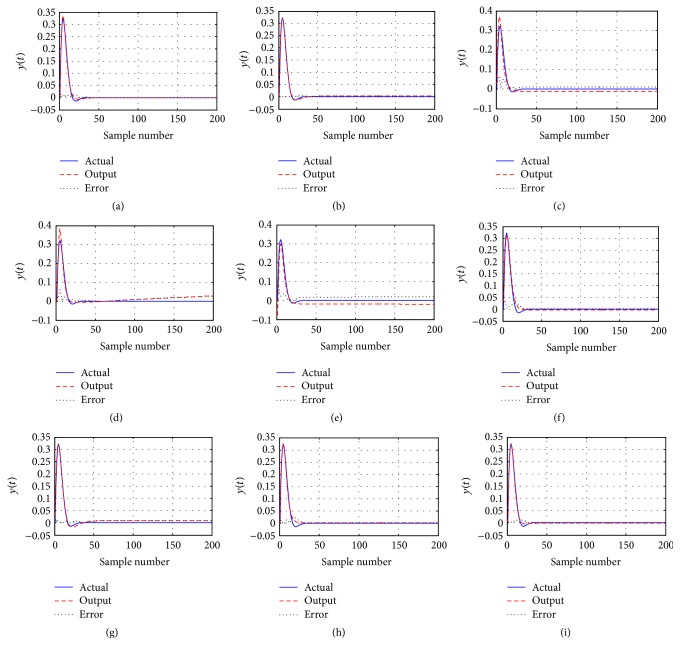
Comparison of the performance curves using different algorithm. (a)~(i) Function approximation with noise using PSO, PSOcf, TLBO, DE, JADE, CMA-ES, ABC, BBO, and CPDD.

Table 9 shows that CPDD performs better than other compared algorithms in terms of the mean MSE and the standard deviation of MSE. The approximation curves for training and test using different algorithms are shown in Figure 6. It indicates that CPDD outperforms other algorithms for training the model.

### 4.2. Lorenz Chaotic Time Series Prediction

In this example, there are three input units, five hidden units, and one output unit in the three-layer feed-forward ANN. It is used to forecast a Lorenz chaotic time series which is described by the following equation [51]:

(9) dxdt=ay−xdydt=bx−xz+cydzdt=xy−dz,

where *a* = 10, *b* = 28, and *c* = 8/3.

In order to train the ANN, 10000 pieces of data are selected according to the real model, among which the first 8000 pieces of data are discarded; the rest normalized 2000 pieces of data are selected as experiment data. The former 1500 points are chosen as the training data points, and the rest 500 points are chosen as the testing data points to test the validity of the model. The simulation goal is to build the single-step-ahead prediction model of chaotic time series as the following form [51]:

(10) $$\begin{array}{c} \hat{x}\left(\;t+1\;\right)=f\left(\;x\left(\;t-6\;\right),x\left(\;t-3\;\right),x\left(\;t\;\right)\;\right). \end{array}$$

In our experiment, population size is set as 50; the maximal number of function evaluations (FES = 10000) is used as ended condition of each algorithm. The results are shown in Table 10 in terms of the mean MSE and the standard deviation obtained in the 50 independent runs for nine optimization algorithms.

**Figure 7:**
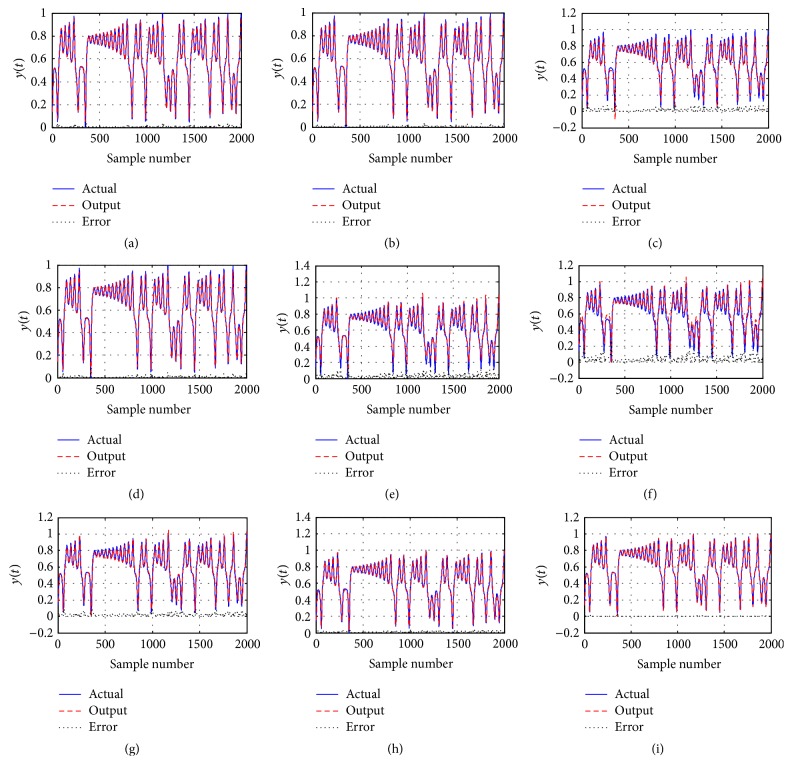
Comparison of the performance curves using different algorithm. (a)~(i) Time series prediction using PSO, PSOcf, TLBO, DE, JADE, CMA-ES, ABC, BBO, and CPDD.

Table 10 indicates that CPDD shows better performance than other compared methods in terms of the mean MSE and the standard deviation.  Figure 7 shows the prediction of Lorenz chaotic time series for training and test with different optimization algorithm. The curves show that the prediction obtained by CPDD performs better than other algorithms. The experimental results represent that CPDD has better prediction performance for Lorenz chaotic time series compared with other optimization algorithms.

## 5. Conclusion

A new metaheuristic optimization algorithm CPDD, which is inspired from the phenomenon of cloud transformation and the transition of matter state, is proposed in this paper. Data-driven mechanism is introduced into differential cloud particles evolution algorithm and it is applied to 30 benchmark problems from the CEC2014 Special Session on Real-Parameter Single Objective Optimization benchmark suite. The experimental results showed that phase transition and data-driven mechanism can not only balance the exploration and exploitation capacity of CPDD but also accelerate the convergence rate. It can be also concluded that CPDD shows better performance on artificial neural network training compared with other optimization algorithms.

However, these methods show poor performance on composite problems. This indicates that the control parameters produced by data-driven mechanism are not appropriate for these problems. Therefore, the exploitation of CPDD is poor on these problems. How to improve the exploitation ability of CPDD will need to be further gone into. It is necessary to introduce some new techniques in CPDD for improving exploration and exploitation ability to solve hybrid composition problems. In addition, CPDD will be used to solve real-world engineering problems.

## Figures and Tables

**Algorithm 1:**
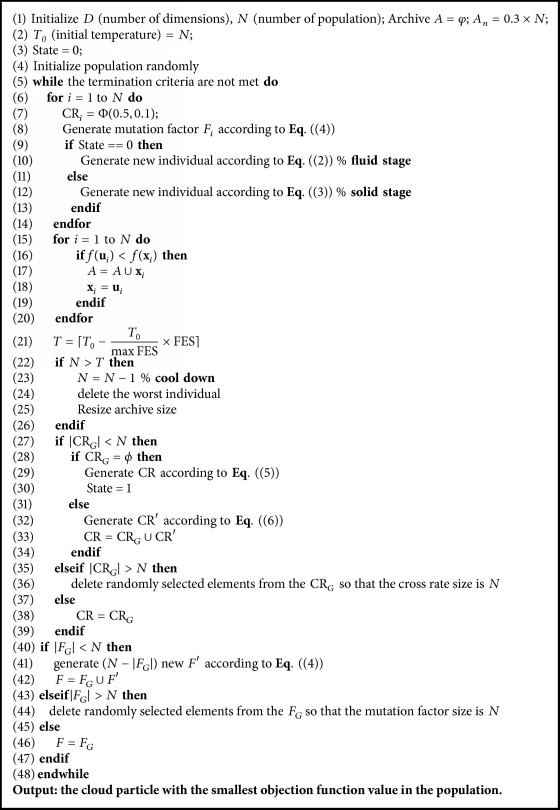
CPDD algorithm.

**Table 1:** Experimental results of PSO, PSOcf, TLBO, DE, JADE, CMA-ES, ABC, BBO, and CPDD on *f*_1_–*f*_3_ test problems of 10 and 30 variables.

*F*(*D* = 10)		PSO	PSOcf	TLBO	DE	JADE	CMA-ES	ABC	BBO	CPDD
*f*_1_	*F*_mean_	7.05*e* + 03(+)	4.36*e* + 04(+)	5.42*e* + 05(+)	1.42*e* − 14(+)	0.00**e** + 00(≈)	0.00**e** + 00(≈)	1.93*e* + 05(+)	4.82*e* + 05(+)	0.00**e** + 00
	SD	1.50*e* + 04	2.78*e* + 04	7.53*e* + 05	2.90*e* − 14	0.00**e** + 00	0.00**e** + 00	1.52*e* + 05	7.59*e* + 05	0.00**e** + 00
	Max	1.38*e* + 05	1.06*e* + 05	2.35*e* + 06	1.27*e* − 13	0.00**e** + 00	0.00**e** + 00	5.81*e* + 05	3.25*e* + 06	0.00**e** + 00
	Min	1.76*e* + 02	4.67*e* + 03	2.18*e* + 03	0.00**e** + 00	0.00**e** + 00	0.00**e** + 00	1.78*e* + 04	1.99*e* + 04	0.00**e** + 00

*f*_2_	*F*_mean_	2.86*e* + 03(+)	2.65*e* + 02(+)	6.05*e* + 03(+)	0.00**e** + 00(≈)	0.00**e** + 00(≈)	0.00**e** + 00(≈)	8.01*e* + 01(+)	2.82*e* + 04(+)	0.00**e** + 00
	SD	2.38*e* + 03	4.53*e* + 02	4.21*e* + 03	0.00**e** + 00	0.00**e** + 00	0.00**e** + 00	8.73*e* + 01	2.48*e* + 04	0.00**e** + 00
	Max	7.77*e* + 03	2.18*e* + 03	1.18*e* + 04	0.00**e** + 00	0.00**e** + 00	0.00**e** + 00	3.16*e* + 02	1.14*e* + 05	0.00**e** + 00
	Min	1.03*e* − 01	1.18*e* − 02	4.43*e* + 00	0.00**e** + 00	0.00**e** + 00	0.00**e** + 00	6.22*e* − 02	4.70*e* + 03	0.00**e** + 00

*f*_3_	*F*_mean_	4.03*e* + 02(+)	1.05*e* + 03(+)	2.30*e* + 02(+)	0.00**e** + 00(≈)	5.31*e* − 03(+)	0.00**e** + 00(≈)	2.95*e* + 02(+)	6.05*e* + 03(+)	0.00**e** + 00
	SD	4.33*e* + 02	6.08*e* + 02	2.84*e* + 02	0.00**e** + 00	1.24*e* − 02	0.00**e** + 00	3.12*e* + 02	5.66*e* + 03	0.00**e** + 00
	Max	1.83*e* + 03	3.25*e* + 03	8.67*e* + 02	0.00**e** + 00	5.38*e* − 02	0.00**e** + 00	1.43*e* + 03	2.36*e* + 04	0.00**e** + 00
	Min	2.73*e* + 01	1.63*e* + 02	7.03*e* − 01	0.00**e** + 00	0.00**e** + 00	0.00**e** + 00	1.76*e* + 01	1.11*e* + 02	0.00**e** + 00

*F*(*D* = 30)		PSO	PSOcf	TLBO	DE	JADE	CMA-ES	ABC	BBO	CPDD

*f*_1_	*F*_mean_	7.54*e* + 06(+)	4.71*e* + 05(+)	5.91*e* + 07(+)	7.20*e* + 04(+)	4.94*e* + 02(+)	1.42**e** − 14(−)	1.05*e* + 07(+)	1.88*e* + 07(+)	4.37*e* − 05
	SD	8.72*e* + 06	4.59*e* + 05	5.69*e* + 07	5.47*e* + 04	9.95*e* + 02	2.02**e** − 14	2.71*e* + 06	1.12*e* + 07	2.68*e* − 05
	Max	3.47*e* + 07	1.64*e* + 06	2.55*e* + 08	2.11*e* + 05	4.23*e* + 03	5.68**e** − 14	1.77*e* + 07	4.90*e* + 07	9.79*e* − 05
	Min	1.45*e* + 05	8.90*e* + 04	4.42*e* + 06	5.19*e* + 03	3.01*e* − 02	1.42**e** − 14	5.98*e* + 06	3.23*e* + 06	5.87*e* − 06

*f*_2_	*F*_mean_	5.22*e* − 01(+)	1.32*e* + 01(+)	8.95*e* + 09(+)	0.00*e* + 00(≈)	0.00*e* + 00(≈)	2.84*e* − 14(+)	2.49*e* + 02(+)	4.23*e* + 06(+)	0.00**e** + 00
	SD	1.19*e* + 00	1.35*e* + 01	7.54*e* + 09	7.46*e* − 15	2.17*e* − 14	3.87*e* − 14	2.56*e* + 02	1.87*e* + 06	0.00**e** + 00
	Max	5.01*e* + 00	5.85*e* + 01	4.09*e* + 10	2.84*e* − 14	2.84*e* − 14	1.13*e* − 13	1.11*e* + 03	9.53*e* + 06	0.00**e** + 00
	Min	2.75*e* − 07	1.51*e* + 00	1.12*e* + 09	0.00*e* + 00	0.00*e* + 00	2.84*e* − 14	1.16*e* + 00	1.68*e* + 06	0.00**e** + 00

*f*_3_	*F*_mean_	3.92*e* + 00(+)	6.14*e* + 02(+)	3.11*e* + 03(+)	0.00*e* + 00(≈)	7.53*e* − 03(+)	5.68*e* − 14(+)	9.44*e* + 02(+)	6.73*e* + 03(+)	0.00**e** + 00
	SD	6.44*e* + 00	4.16*e* + 02	6.40*e* + 03	1.05*e* − 14	3.50*e* − 02	6.67*e* − 14	7.89*e* + 02	5.92*e* + 03	0.00**e** + 00
	Max	2.82*e* + 01	1.69*e* + 03	2.71*e* + 04	5.68*e* − 14	1.90*e* − 01	2.27*e* − 13	2.98*e* + 03	2.12*e* + 04	0.00**e** + 00
	Min	7.95*e* − 03	3.50*e* + 01	5.25*e* − 02	0.00*e* + 00	5.68*e* − 14	5.68*e* − 14	3.59*e* + 01	3.75*e* + 01	0.00**e** + 00

**Table 2:** Experimental results of PSO, PSOcf, TLBO, DE, JADE, CMA-ES, ABC, BBO, and CPDD on *f*_4_–*f*_16_ test problems of 10 variables.

*F*		PSO	PSOcf	TLBO	DE	JADE	CMA-ES	ABC	BBO	CPDD
*f*_4_	*F*_mean_	2.19*e* + 01(−)	3.33*e* + 01(≈)	1.82*e* + 01(−)	1.98*e* + 01(−)	3.02*e* + 01(≈)	2.91*e* + 01(≈)	1.87**e** − 01(−)	2.04*e* + 01(−)	3.26*e* + 01
	SD	1.84*e* + 01	2.00*e* + 01	1.68*e* + 01	1.73*e* + 01	1.16*e* + 01	1.28*e* + 01	3.41**e** − 01	1.87*e* + 01	8.29*e* + 00
	Max	4.06*e* + 01	1.33*e* + 02	3.47*e* + 01	3.47*e* + 01	3.47*e* + 01	3.47*e* + 01	1.80**e** + 00	6.46*e* + 01	3.47*e* + 01
	Min	6.69*e* − 03	1.79*e* + 01	6.53*e* − 06	0.00**e** + 00	0.00**e** + 00	0.00**e** + 00	5.67*e* − 03	7.41*e* − 03	0.00**e** + 00

*f*_5_	*F*_mean_	2.02*e* + 01(+)	2.00*e* + 01(+)	1.91*e* + 01(+)	2.03*e* + 01(+)	1.70*e* + 01(+)	1.99*e* + 01(+)	1.83*e* + 01(+)	1.93*e* + 01(+)	8.89**e** − 01
	SD	8.33*e* − 02	9.03*e* − 02	4.53*e* + 00	7.21*e* − 02	4.51*e* + 00	3.75**e** − 05	5.02*e* + 00	3.59*e* + 00	1.37*e* + 00
	Max	2.04*e* + 01	2.02*e* + 01	2.04*e* + 01	2.04*e* + 01	2.00*e* + 01	1.99*e* + 01	2.00*e* + 01	2.00*e* + 01	4.55**e** + 00
	Min	2.00*e* + 01	1.99*e* + 01	3.45*e* − 06	2.01*e* + 01	6.52*e* + 00	1.99*e* + 01	3.31*e* + 00	3.33*e* − 01	0.00**e** + 00

*f*_6_	*F*_mean_	1.35*e* + 00(+)	2.43*e* + 00(+)	3.50*e* − 01(+)	5.96*e* − 02(+)	1.53*e* − 01(+)	1.37*e* + 01(+)	2.28*e* + 00(+)	2.26*e* + 00(+)	0.00**e** + 00
	SD	1.18*e* + 00	1.68*e* + 00	4.21*e* − 01	1.26*e* − 01	1.97*e* − 01	3.75*e* + 00	4.86*e* − 01	1.05*e* + 00	0.00**e** + 00
	Max	4.24*e* + 00	6.53*e* + 00	1.23*e* + 00	8.94*e* − 01	8.30*e* − 01	1.91*e* + 01	3.17*e* + 00	5.13*e* + 00	0.00**e** + 00
	Min	9.88*e* − 03	6.77*e* − 05	1.13*e* − 13	0.00**e** + 00	1.91*e* − 05	4.10*e* + 00	1.16*e* + 00	8.78*e* − 01	0.00**e** + 00

*f*_7_	*F*_mean_	1.24*e* − 01(+)	4.27*e* + 00(+)	4.42*e* − 02(+)	2.51*e* − 01(+)	9.72*e* − 03(+)	1.06*e* − 02(+)	8.49*e* − 03(+)	2.91*e* − 01(+)	0.00**e** + 00
	SD	5.91*e* − 02	4.77*e* + 00	2.46*e* − 02	1.17*e* − 01	6.53*e* − 03	1.00*e* − 02	8.80*e* − 03	1.51*e* − 01	0.00**e** + 00
	Max	2.53*e* − 01	1.78*e* + 01	9.34*e* − 02	4.23*e* − 01	2.88*e* − 02	3.20*e* − 02	3.21*e* − 02	8.21*e* − 01	0.00**e** + 00
	Min	1.72*e* − 02	4.42*e* − 02	7.39*e* − 03	8.87*e* − 03	4.91*e* − 04	0.00**e** + 00	2.16*e* − 12	1.15*e* − 01	0.00**e** + 00

*f*_8_	*F*_mean_	1.59*e* + 00(+)	7.53*e* + 00(+)	5.45*e* + 00(+)	1.64*e* + 01(+)	0.00**e** + 00(≈)	1.47*e* + 02(+)	0.00**e** + 00(≈)	6.54*e* − 03(+)	0.00**e** + 00
	SD	9.63*e* − 01	3.72*e* + 00	2.89*e* + 00	2.72*e* + 00	0.00**e** + 00	4.59*e* + 01	2.11*e* − 14	3.57*e* − 03	0.00**e** + 00
	Max	3.97*e* + 00	1.62*e* + 01	1.73*e* + 01	2.19*e* + 01	0.00**e** + 00	2.58*e* + 02	1.13*e* − 13	1.39*e* − 02	0.00**e** + 00
	Min	0.00**e** + 00	1.98*e* + 00	9.94*e* − 01	8.44*e* + 00	0.00**e** + 00	6.96*e* + 01	0.00**e** + 00	1.01*e* − 03	0.00**e** + 00

*f*_9_	*F*_mean_	7.94*e* + 00(+)	1.34*e* + 01(+)	6.65*e* + 00(+)	2.39*e* + 01(+)	3.30*e* + 00(+)	1.51*e* + 02(+)	8.43*e* + 00(+)	8.44*e* + 00(+)	2.70**e** + 00
	SD	3.58*e* + 00	5.08*e* + 00	2.72*e* + 00	4.05*e* + 00	7.48*e* − 01	4.79*e* + 01	1.88*e* + 00	2.82*e* + 00	6.77**e** − 01
	Max	1.49*e* + 01	2.38*e* + 01	1.29*e* + 01	3.19*e* + 01	4.84*e* + 00	3.24*e* + 02	1.13*e* + 01	1.40*e* + 01	3.44**e** + 00
	Min	1.98*e* + 00	3.97*e* + 00	2.56*e* + 00	1.57*e* + 01	1.36*e* + 00	7.16*e* + 01	4.01*e* + 00	2.00*e* + 00	1.00**e** + 00

*f*_10_	*F*_mean_	1.28*e* + 02(+)	2.73*e* + 02(+)	2.20*e* + 02(+)	6.24*e* + 02(+)	4.16**e** − 03(−)	1.76*e* + 03(+)	9.71*e* − 02(−)	1.48*e* − 01(−)	3.64*e* + 00
	SD	9.69*e* + 01	2.06*e* + 02	1.87*e* + 02	1.54*e* + 02	1.58**e** − 02	4.62*e* + 02	5.68*e* − 02	6.08*e* − 02	2.31*e* + 00
	Max	3.75*e* + 02	7.02*e* + 02	8.83*e* + 02	8.90*e* + 02	6.24**e** − 02	3.12*e* + 03	1.91*e* − 01	2.79*e* − 01	6.95*e* + 00
	Min	1.34*e* + 01	1.57*e* + 01	1.03*e* + 01	2.74*e* + 02	0.00**e** + 00	1.03*e* + 03	1.56*e* − 07	3.56*e* − 02	1.87*e* − 01

*f*_11_	*F*_mean_	2.62*e* + 02(+)	4.04*e* + 02(+)	8.35*e* + 02(+)	1.04*e* + 03(+)	1.05**e** + 02(≈)	1.47*e* + 03(+)	2.11*e* + 02(+)	2.21*e* + 02(+)	1.08*e* + 02
	SD	2.24*e* + 02	2.09*e* + 02	2.18*e* + 02	1.46*e* + 02	6.78**e** + 01	3.69*e* + 02	8.70*e* + 01	1.32*e* + 02	8.18*e* + 01
	Max	9.37*e* + 02	7.63*e* + 02	1.36*e* + 03	1.33*e* + 03	2.98*e* + 02	2.20*e* + 03	3.42*e* + 02	5.26*e* + 02	2.73**e** + 02
	Min	1.84*e* + 01	2.40*e* + 01	3.27*e* + 02	7.30*e* + 02	3.19*e* + 01	8.27*e* + 02	4.76*e* + 01	1.61*e* + 01	9.76**e** + 00

*f*_12_	*F*_mean_	4.26*e* − 01(+)	1.06**e** − 01(−)	1.09*e* + 00(+)	9.01*e* − 01(+)	2.53*e* − 01(−)	1.02*e* + 00(+)	2.70*e* − 01(≈)	1.34*e* − 01(−)	2.90*e* − 01
	SD	3.23*e* − 01	5.10**e** − 02	2.30*e* − 01	1.28*e* − 01	6.31*e* − 02	1.31*e* + 00	5.87*e* − 02	5.59*e* − 02	7.30*e* − 02
	Max	1.15*e* + 00	2.06**e** − 01	1.45*e* + 00	1.16*e* + 00	4.28*e* − 01	4.35*e* + 00	3.50*e* − 01	2.71*e* − 01	3.99*e* − 01
	Min	2.15*e* − 02	1.70*e* − 02	5.38*e* − 01	6.86*e* − 01	1.60*e* − 01	1.09**e** − 02	1.52*e* − 01	4.86*e* − 02	8.60*e* − 02

*f*_13_	*F*_mean_	1.29*e* − 01(+)	2.81*e* − 01(+)	1.38*e* − 01(+)	1.45*e* − 01(+)	8.64*e* − 02(+)	1.09*e* − 01(+)	1.19*e* − 01(+)	2.82*e* − 01(+)	7.38**e** − 02
	SD	5.81*e* − 02	8.69*e* − 02	4.07*e* − 02	2.62*e* − 02	1.54**e** − 02	4.51*e* − 02	2.34*e* − 02	5.85*e* − 02	2.33**e** − 02
	Max	2.39*e* − 01	4.49*e* − 01	2.22*e* − 01	2.01*e* − 01	1.13*e* − 01	2.27*e* − 01	1.79*e* − 01	4.16*e* − 01	1.01**e** − 01
	Min	3.33*e* − 02	9.48*e* − 02	6.40*e* − 02	1.02*e* − 01	5.81*e* − 02	2.85*e* − 02	5.86*e* − 02	1.50*e* − 01	2.15**e** − 02

*f*_14_	*F*_mean_	1.33*e* − 01(+)	4.65*e* − 01(+)	2.66*e* − 01(+)	1.65*e* − 01(+)	9.72*e* − 02(+)	4.56*e* − 01(+)	1.39*e* − 01(+)	2.56*e* − 01(+)	6.70**e** − 02
	SD	4.71*e* − 02	3.69*e* − 01	6.42*e* − 02	3.14*e* − 02	2.63*e* − 02	5.36*e* − 02	2.74*e* − 02	1.44*e* − 01	1.98**e** − 02
	Max	2.37*e* − 01	1.00*e* + 00	4.19*e* − 01	2.26*e* − 01	1.47*e* − 01	4.99*e* − 01	2.05*e* − 01	7.41*e* − 01	9.56**e** − 02
	Min	5.09*e* − 02	7.10*e* − 02	1.66*e* − 01	8.90*e* − 02	5.36*e* − 02	2.86*e* − 01	7.90*e* − 02	9.61*e* − 02	2.03**e** − 02

*f*_15_	*F*_mean_	8.47*e* − 01(≈)	2.31*e* + 00(≈)	1.40*e* + 00(+)	1.99*e* + 00(+)	5.52**e** − 01(−)	1.00*e* + 00(+)	9.36*e* − 01(+)	1.81*e* + 00(+)	7.98*e* − 01
	SD	3.90*e* − 01	7.84*e* + 00	3.27*e* − 01	3.92*e* − 01	8.77**e** − 02	3.29*e* − 01	2.18*e* − 01	6.48*e* − 01	1.29**e** − 01
	Max	2.49*e* + 00	4.37*e* + 01	1.97*e* + 00	2.54*e* + 00	7.25**e** − 01	1.78*e* + 00	1.33*e* + 00	3.29*e* + 00	9.55**e** − 01
	Min	3.94*e* − 01	4.31*e* − 01	8.44*e* − 01	8.23*e* − 01	3.37**e** − 01	4.36*e* − 01	2.62**e** − 01	6.42*e* − 01	5.15*e* − 01

*f*_16_	*F*_mean_	2.16*e* + 00(+)	2.39*e* + 00(+)	2.39*e* + 00(+)	2.76*e* + 00(+)	1.60*e* + 00(+)	4.72*e* + 00(+)	2.23*e* + 00(+)	2.25*e* + 00(+)	1.00**e** + 00
	SD	4.58*e* − 01	4.58*e* − 01	2.77*e* − 01	1.94*e* − 01	2.44*e* − 01	1.73**e** − 01	2.40*e* − 01	4.37*e* − 01	1.83*e* − 01
	Max	3.06*e* + 00	3.23*e* + 00	2.95*e* + 00	3.07*e* + 00	2.10*e* + 00	4.97*e* + 00	2.63*e* + 00	2.85*e* + 00	1.22**e** + 00
	Min	1.43*e* + 00	1.54*e* + 00	1.44*e* + 00	2.20*e* + 00	9.74*e* − 01	4.22*e* + 00	1.72*e* + 00	9.31*e* − 01	5.36**e** − 01

**Table 3:** Experimental results of PSO, PSOcf, TLBO, DE, JADE, CMA-ES, ABC, BBO, and CPDD on *f*_4_–*f*_16_ test problems of 30 variables.

*F*		PSO	PSOcf	TLBO	DE	JADE	CMA-ES	ABC	BBO	CPDD
*f*_4_	*F*_mean_	1.66*e* + 02(+)	5.41*e* + 02(+)	5.41*e* + 01(+)	1.16*e* − 01(+)	1.32*e* − 01(≈)	2.11*e* + 00(≈)	1.81*e* + 01(+)	1.29*e* + 02(+)	3.57**e** − 05
	SD	4.11*e* + 01	6.37*e* + 02	3.11*e* + 01	7.41*e* − 02	7.27*e* − 01	1.15*e* + 01	2.50*e* + 01	3.44*e* + 01	8.95**e** − 05
	Max	2.99*e* + 02	3.18*e* + 03	9.54*e* + 01	3.57*e* − 01	3.98*e* + 00	6.34*e* + 01	7.23*e* + 01	1.95*e* + 02	3.60**e** − 04
	Min	7.73*e* + 01	7.74*e* + 01	2.54*e* − 01	1.54*e* − 03	5.68**e** − 14	5.68**e** − 14	3.66*e* − 01	7.67*e* + 01	6.36*e* − 12

*f*_5_	*F*_mean_	2.07*e* + 01(+)	2.02*e* + 01(−)	2.09*e* + 01(+)	2.09*e* + 01(+)	2.02*e* + 01(−)	1.99**e** + 01(−)	2.03*e* + 01(−)	2.01*e* + 01(−)	2.04*e* + 01
	SD	1.29*e* − 01	2.72*e* − 01	8.08*e* − 02	5.87*e* − 02	3.57*e* − 02	1.01**e** − 05	2.57*e* − 02	4.88*e* − 02	7.76*e* − 02
	Max	2.09*e* + 01	2.08*e* + 01	2.09*e* + 01	2.10*e* + 01	2.03*e* + 01	1.99**e** + 01	2.04*e* + 01	2.02*e* + 01	2.05*e* + 01
	Min	2.04*e* + 01	1.99*e* + 01	2.06*e* + 01	2.07*e* + 01	2.02*e* + 01	1.99**e** + 01	2.02*e* + 01	2.00*e* + 01	2.02*e* + 01

*f*_6_	*F*_mean_	1.27*e* + 01(+)	1.93*e* + 01(+)	1.21*e* + 01(+)	2.96*e* − 01(+)	9.09*e* + 00(+)	4.29*e* + 01(+)	1.50*e* + 01(+)	1.28*e* + 01(+)	0.00**e** + 00
	SD	3.19*e* + 00	4.07*e* + 00	2.27*e* + 00	5.63*e* − 01	3.32*e* + 00	8.41*e* + 00	1.48*e* + 00	2.01*e* + 00	0.00**e** + 00
	Max	2.04*e* + 01	2.67*e* + 01	1.68*e* + 01	1.84*e* + 00	1.28*e* + 01	5.79*e* + 01	1.66*e* + 01	1.67*e* + 01	0.00**e** + 00
	Min	6.84*e* + 00	1.02*e* + 01	8.15*e* + 00	1.13*e* − 13	0.00*e* + 00	1.97*e* + 01	1.06*e* + 01	9.22*e* + 00	0.00**e** + 00

*f*_7_	*F*_mean_	1.08*e* − 02(+)	7.30*e* + 01(+)	6.11*e* − 02(+)	0.00**e** + 00(≈)	0.00**e** + 00(≈)	1.56*e* − 03(+)	9.52*e* − 07(+)	1.04*e* + 00(+)	0.00**e** + 00
	SD	1.32*e* − 02	4.48*e* + 01	2.29*e* − 01	0.00**e** + 00	4.72*e* − 14	4.06*e* − 03	1.82*e* − 06	1.78*e* − 02	0.00**e** + 00
	Max	4.42*e* − 02	2.01*e* + 02	1.26*e* + 00	0.00**e** + 00	1.13*e* − 13	1.23*e* − 02	6.48*e* − 06	1.07*e* + 00	0.00**e** + 00
	Min	3.41*e* − 13	1.49*e* + 01	5.68*e* − 13	0.00**e** + 00	0.00**e** + 00	1.13*e* − 13	2.01*e* − 11	9.95*e* − 01	0.00**e** + 00

*f*_8_	*F*_mean_	2.99*e* + 01(+)	8.25*e* + 01(+)	5.74*e* + 01(+)	1.36*e* + 02(+)	0.00**e** + 00(−)	4.23*e* + 02(+)	4.54*e* − 13(−)	5.55*e* − 01(−)	7.35*e* + 00
	SD	6.08*e* + 00	2.79*e* + 01	1.15*e* + 01	2.49*e* + 01	0.00**e** + 00	7.73*e* + 01	6.62*e* − 13	2.35*e* − 01	2.25*e* + 00
	Max	4.27*e* + 01	1.68*e* + 02	7.95*e* + 01	1.80*e* + 02	0.00**e** + 00	6.03*e* + 02	2.95*e* − 12	1.11*e* + 00	1.05*e* + 01
	Min	2.08*e* + 01	4.79*e* + 01	3.78*e* + 01	8.55*e* + 01	0.00**e** + 00	3.10*e* + 02	2.27*e* − 13	1.82*e* − 01	2.98*e* + 00

*f*_9_	*F*_mean_	6.64*e* + 01(+)	1.11*e* + 02(+)	6.31*e* + 01(+)	1.75*e* + 02(+)	2.56**e** + 01(−)	6.34*e* + 02(+)	8.68*e* + 01(+)	6.01*e* + 01(+)	3.14*e* + 01
	SD	1.49*e* + 01	3.55*e* + 01	1.32*e* + 01	1.08*e* + 02	4.50**e** + 00	9.84*e* + 01	1.30*e* + 01	1.75*e* + 01	6.41*e* + 00
	Max	1.00*e* + 02	1.76*e* + 02	9.45*e* + 01	1.92*e* + 01	3.59**e** + 01	8.16*e* + 02	1.09*e* + 02	1.05*e* + 02	3.99*e* + 01
	Min	4.17*e* + 01	3.48*e* + 01	4.17*e* + 01	1.52*e* + 02	1.72**e** + 01	4.09*e* + 02	6.44*e* + 01	2.99*e* + 01	1.79*e* + 01

*f*_10_	*F*_mean_	8.20*e* + 02(+)	2.30*e* + 03(+)	1.23*e* + 03(+)	5.48*e* + 03(+)	7.63**e** − 03(−)	4.86*e* + 03(+)	1.35*e* + 00(−)	3.85*e* + 00(−)	3.75*e* + 01
	SD	2.93*e* + 02	5.85*e* + 02	8.55*e* + 02	5.86*e* + 02	1.28*e* − 02	8.99*e* + 02	7.19*e* − 01	1.02*e* + 00	2.15*e* + 01
	Max	1.39*e* + 03	3.73*e* + 03	4.40*e* + 03	6.26*e* + 03	4.16*e* − 02	7.19*e* + 03	3.58*e* + 00	6.25*e* + 00	7.96*e* + 01
	Min	2.53*e* + 02	1.21*e* + 03	2.41*e* + 02	6.26*e* + 03	0.00**e** + 00	2.84*e* + 03	2.01*e* − 01	2.26*e* + 00	6.99*e* + 00

*f*_11_	*F*_mean_	2.87*e* + 03(+)	3.42*e* + 03(+)	6.55*e* + 03(+)	6.67*e* + 03(+)	1.70**e** + 03(−)	4.95*e* + 03(+)	2.21*e* + 03(−)	2.06*e* + 03(−)	2.54*e* + 03
	SD	5.83*e* + 02	6.26*e* + 02	3.06*e* + 02	2.60*e* + 02	2.16*e* + 02	8.66*e* + 02	1.87**e** + 02	3.63*e* + 02	3.62*e* + 02
	Max	4.01*e* + 03	4.92*e* + 03	6.99*e* + 03	7.13*e* + 03	2.04**e** + 03	6.81*e* + 03	2.56*e* + 03	2.79*e* + 03	3.11*e* + 03
	Min	1.62**e** + 03	2.18*e* + 03	5.49*e* + 03	6.14*e* + 03	1.08**e** + 03	3.03*e* + 03	1.70*e* + 03	1.08*e* + 03	1.86*e* + 03

*f*_12_	*F*_mean_	5.86*e* − 01(+)	2.72*e* − 01(+)	2.50*e* + 00(+)	2.51*e* + 00(+)	2.73*e* − 01(+)	3.08*e* − 01(≈)	4.12*e* − 01(+)	2.34*e* − 01(≈)	2.22**e** − 01
	SD	2.32*e* − 01	9.25*e* − 02	2.50*e* − 01	3.19*e* − 01	3.51**e** − 02	2.62*e* − 01	5.55*e* − 02	5.74*e* − 02	5.43*e* − 02
	Max	1.12*e* + 00	4.39*e* − 01	2.91*e* + 00	3.00*e* + 00	3.32*e* − 01	1.36*e* + 00	4.95*e* − 01	3.48*e* − 01	3.18**e** − 01
	Min	2.46*e* − 01	1.21*e* − 01	1.87*e* + 00	1.69*e* + 00	1.77*e* − 01	4.15**e** − 02	3.02*e* − 01	1.06*e* − 01	1.44*e* − 01

*f*_13_	*F*_mean_	4.02*e* − 01(+)	1.23*e* + 00(+)	3.87*e* − 01(+)	3.51*e* − 01(+)	2.16*e* − 01(≈)	2.37*e* − 01(+)	2.44*e* − 01(+)	5.91*e* − 01(+)	2.01**e** − 01
	SD	9.77*e* − 02	9.35*e* − 01	7.57*e* − 02	5.13*e* − 02	3.86*e* − 02	7.37*e* − 02	2.55**e** − 02	1.05*e* − 01	5.11*e* − 02
	Max	5.74*e* − 01	3.81*e* + 00	5.61*e* − 01	4.74*e* − 01	3.34*e* − 01	4.22*e* − 01	2.87*e* − 01	8.46*e* − 01	2.73**e** − 01
	Min	2.46*e* − 01	3.44*e* − 01	2.35*e* − 01	2.67*e* − 01	1.57*e* − 01	1.27*e* − 01	1.98*e* − 01	4.00*e* − 01	9.08**e** − 02

*f*_14_	*F*_mean_	2.98*e* − 01(+)	2.41*e* + 01(+)	2.72*e* − 01(+)	2.76*e* − 01(+)	2.39*e* − 01(+)	3.62*e* − 01(+)	1.94**e** − 01(−)	3.94*e* − 01(+)	2.18*e* − 01
	SD	1.40*e* − 01	2.12*e* + 01	5.02*e* − 02	2.85*e* − 02	2.34*e* − 02	1.12*e* − 01	1.17**e** − 02	1.76*e* − 01	2.61*e* − 02
	Max	7.86*e* − 01	7.11*e* + 01	3.69*e* − 01	3.28*e* − 01	2.88*e* − 01	8.92*e* − 01	2.17**e** − 01	1.07*e* + 00	2.53*e* − 01
	Min	1.70*e* − 01	3.23*e* − 01	1.91*e* − 01	2.27*e* − 01	2.06*e* − 01	2.43*e* − 01	1.75*e* − 01	2.69*e* − 01	1.56**e** − 01

*f*_15_	*F*_mean_	8.34*e* + 00(+)	1.05*e* + 03(≈)	9.80*e* + 00(+)	1.57*e* + 01(+)	3.10**e** + 00(−)	3.74*e* + 00(−)	1.05*e* + 01(+)	1.44*e* + 01(+)	4.94*e* + 00
	SD	3.68*e* + 00	3.61*e* + 03	4.11*e* + 00	8.13*e* − 01	3.37**e** − 01	9.59*e* − 01	1.57*e* + 00	3.54*e* + 00	9.54*e* − 01
	Max	2.08*e* + 01	1.97*e* + 04	2.09*e* + 01	1.74*e* + 01	3.90**e** + 00	5.95**e** + 00	1.32*e* + 01	2.13*e* + 01	6.19*e* + 00
	Min	4.11*e* + 00	2.58*e* + 00	3.19*e* + 00	1.41*e* + 01	2.27**e** + 00	2.21**e** + 00	6.51*e* + 00	8.45*e* + 00	2.49*e* + 00

*f*_16_	*F*_mean_	1.09*e* + 01(+)	1.10*e* + 01(+)	1.17*e* + 01(+)	1.24*e* + 01(+)	9.35**e** + 00(−)	1.43*e* + 01(+)	1.06*e* + 01(≈)	9.53*e* + 00(−)	1.03*e* + 01
	SD	5.57*e* − 01	6.72*e* − 01	4.21*e* − 01	2.05*e* − 01	3.37*e* − 01	3.32*e* − 01	2.98**e** − 01	7.71*e* − 01	7.86*e* − 01
	Max	1.18*e* + 01	1.25*e* + 01	1.24*e* + 01	1.28*e* + 01	9.97**e** + 00	1.47*e* + 01	1.10*e* + 01	1.07*e* + 01	1.14*e* + 01
	Min	9.58*e* + 00	9.54*e* + 00	1.06*e* + 01	1.20*e* + 01	8.67*e* + 00	1.35*e* + 01	9.61*e* + 00	6.94*e* + 00	8.35**e** + 00

**Table 4:** Experimental results of PSO, PSOcf, TLBO, DE, JADE, CMA-ES, ABC, BBO, and CPDD on *f*_17_–*f*_22_ test problems of 10 variables.

*F*		PSO	PSOcf	TLBO	DE	JADE	CMA-ES	ABC	BBO	CPDD
*f*_17_	*F*_mean_	3.20*e* + 03(+)	5.38*e* + 03(+)	1.49*e* + 03(+)	5.78**e** − 01(≈)	1.85*e* + 00(≈)	5.26*e* + 02(+)	1.74*e* + 05(+)	3.13*e* + 05(+)	9.85*e* − 01
	SD	2.53*e* + 03	4.21*e* + 03	6.26*e* + 02	8.15*e* − 01	2.20*e* + 00	1.94*e* + 02	1.78*e* + 05	4.17*e* + 05	7.30**e** − 01
	Max	9.98*e* + 03	1.50*e* + 04	3.82*e* + 03	4.69*e* + 00	7.04*e* + 00	8.93*e* + 02	6.68*e* + 05	2.02*e* + 06	2.46**e** + 00
	Min	3.33*e* + 02	2.72*e* + 02	6.11*e* + 02	4.37*e* − 10	4.54*e* − 13	1.51*e* + 02	1.03*e* + 04	3.18*e* + 04	2.27**e** − 13

*f*_18_	*F*_mean_	5.63*e* + 03(+)	8.15*e* + 03(+)	1.04*e* + 03(+)	1.92*e* − 01(+)	1.84*e* − 01(+)	5.99*e* + 01(+)	6.90*e* + 02(+)	1.08*e* + 04(+)	5.06**e** − 02
	SD	8.27*e* + 03	6.92*e* + 03	1.18*e* + 03	1.81*e* − 01	3.16*e* − 01	3.49*e* + 01	4.79*e* + 02	1.20*e* + 04	4.05**e** − 02
	Max	2.77*e* + 04	2.72*e* + 04	5.03*e* + 03	4.99*e* − 01	1.70*e* + 00	1.50*e* + 02	1.77*e* + 03	3.68*e* + 04	1.26**e** − 01
	Min	2.95*e* + 00	2.03*e* + 02	7.83*e* + 01	1.72*e* − 02	6.05*e* − 03	4.09*e* + 00	1.17*e* + 02	1.11*e* + 02	1.42**e** − 03

*f*_19_	*F*_mean_	1.14*e* + 00(+)	4.28*e* + 00(+)	1.19*e* + 00(+)	4.65*e* − 01(+)	2.33*e* − 01(+)	2.77*e* + 00(+)	5.35*e* − 01(+)	9.57*e* − 01(+)	2.93**e** − 02
	SD	8.69*e* − 01	1.80*e* + 00	6.07*e* − 01	1.14*e* − 01	6.52*e* − 02	1.13*e* + 00	2.38*e* − 01	4.22*e* − 01	1.69**e** − 02
	Max	2.64*e* + 00	7.82*e* + 00	3.01*e* + 00	7.26*e* − 01	3.73*e* − 01	4.90*e* + 00	1.14*e* + 00	1.73*e* + 00	5.12**e** − 02
	Min	4.40*e* − 02	1.05*e* + 00	2.94*e* − 01	2.90*e* − 01	1.04*e* − 01	1.02*e* + 00	1.49*e* − 01	2.81*e* − 01	2.60**e** − 09

*f*_20_	*F*_mean_	5.23*e* + 02(+)	4.01*e* + 02(+)	1.84*e* + 02(+)	8.51*e* − 02(≈)	3.18*e* − 01(+)	6.22*e* + 01(+)	1.62*e* + 02(+)	6.12*e* + 03(+)	8.39**e** − 02
	SD	8.83*e* + 02	4.97*e* + 02	1.08*e* + 02	1.60*e* − 01	1.00*e* − 01	4.38*e* + 01	1.27*e* + 02	7.26*e* + 03	3.64**e** − 02
	Max	4.31*e* + 03	1.75*e* + 03	4.72*e* + 02	5.55*e* − 01	5.87*e* − 01	1.96*e* + 02	4.25*e* + 02	2.64*e* + 04	1.30**e** − 01
	Min	7.84*e* + 00	4.27*e* + 00	4.41*e* + 01	3.71**e** − 05	8.78*e* − 02	1.02*e* + 01	9.76*e* + 00	4.56*e* + 01	2.96*e* − 02

*f*_21_	*F*_mean_	8.88*e* + 01(+)	2.44*e* + 02(+)	2.13*e* + 02(+)	2.67*e* − 01(+)	1.10*e* + 00(+)	2.95*e* + 02(+)	1.09*e* + 04(+)	5.42*e* + 04(+)	7.11**e** − 02
	SD	5.43*e* + 01	1.98*e* + 02	8.94*e* + 01	2.42*e* − 01	1.20*e* + 00	1.65*e* + 02	1.01*e* + 04	8.48*e* + 04	1.14**e** − 01
	Max	1.83*e* + 02	8.57*e* + 02	4.12*e* + 02	8.12*e* − 01	4.97*e* + 00	6.59*e* + 02	4.48*e* + 04	3.40*e* + 05	3.12**e** − 01
	Min	6.74*e* − 01	1.76*e* + 01	6.29*e* + 01	4.30**e** − 05	1.10*e* − 03	1.79*e* + 01	5.93*e* + 02	4.37*e* + 02	6.58**e** − 05

*f*_22_	*F*_mean_	1.58*e* + 01(+)	3.42*e* + 01(+)	1.66*e* + 01(+)	2.75*e* − 01(+)	1.97*e* − 01(+)	2.25*e* + 02(+)	9.50*e* − 01(+)	7.05*e* + 00(+)	5.08**e** − 02
	SD	8.69*e* + 00	2.35*e* + 01	9.03*e* + 00	2.08*e* − 01	5.75*e* − 02	2.24*e* + 02	1.12*e* + 00	2.22*e* + 01	5.54**e** − 02
	Max	2.20*e* + 01	1.40*e* + 02	2.98*e* + 01	7.47*e* − 01	3.30*e* − 01	7.31*e* + 02	4.91*e* + 00	1.21*e* + 02	2.00**e** − 01
	Min	1.97*e* − 02	2.03*e* + 01	1.25*e* + 00	3.50*e* − 02	1.03*e* − 01	2.01*e* + 01	7.10*e* − 02	3.81*e* − 01	1.30**e** − 03

**Table 5:** Experimental results of PSO, PSOcf, TLBO, DE, JADE, CMA-ES, ABC, BBO, and CPDD on *f*_17_–*f*_22_ test problems of 30 variables.

F		PSO	PSOcf	TLBO	DE	JADE	CMA-ES	ABC	BBO	CPDD
*f*_17_	*F*_mean_	6.01*e* + 05(+)	1.08*e* + 06(+)	1.91*e* + 05(+)	1.35*e* + 03(+)	1.21*e* + 03(+)	1.60*e* + 03(+)	2.82*e* + 06(+)	1.78*e* + 06(+)	1.02**e** + 02
	SD	6.73*e* + 05	1.53*e* + 06	1.71*e* + 05	1.86*e* + 02	3.65*e* + 02	4.21*e* + 02	1.29*e* + 06	1.14*e* + 06	4.30**e** + 01
	Max	3.04*e* + 06	6.58*e* + 06	7.16*e* + 05	1.66*e* + 03	1.88*e* + 03	2.31*e* + 03	6.11*e* + 06	4.26*e* + 06	1.76**e** + 02
	Min	1.96*e* + 04	4.15*e* + 04	3.27*e* + 04	9.02*e* + 02	6.12*e* + 02	7.88*e* + 02	9.93*e* + 05	2.41*e* + 05	3.62**e** + 01

*f*_18_	*F*_mean_	5.59*e* + 03(+)	3.86*e* + 07(+)	2.86*e* + 03(+)	5.47*e* + 01(+)	8.34*e* + 01(+)	1.40*e* + 02(+)	2.58*e* + 03(+)	2.99*e* + 03(+)	6.65**e** + 00
	SD	5.03*e* + 03	1.21*e* + 07	3.62*e* + 03	4.64*e* + 00	2.62*e* + 01	4.68*e* + 01	2.04*e* + 03	2.71*e* + 03	1.63**e** + 00
	Max	2.53*e* + 04	5.03*e* + 08	1.59*e* + 04	6.54*e* + 01	1.26*e* + 02	2.44*e* + 02	7.25*e* + 03	1.12*e* + 04	8.94**e** + 00
	Min	9.78*e* + 01	4.56*e* + 02	8.28*e* + 01	4.58*e* + 01	3.01*e* + 01	6.50*e* + 01	2.20*e* + 02	3.23*e* + 02	3.70**e** + 00

*f*_19_	*F*_mean_	9.13*e* + 00(+)	3.06*e* + 01(+)	1.17*e* + 01(+)	4.43*e* + 00(+)	4.37*e* + 00(+)	9.28*e* + 00(+)	7.06*e* + 00(+)	2.58*e* + 01(+)	2.93**e** + 00
	SD	4.97*e* + 00	3.41*e* + 01	1.07*e* + 01	2.91**e** − 01	5.41*e* − 01	1.60*e* + 00	6.23*e* − 01	2.90*e* + 01	6.13**e** − 01
	Max	3.28*e* + 01	1.09*e* + 02	6.73*e* + 01	5.04*e* + 00	5.78*e* + 00	1.21*e* + 01	8.03*e* + 00	8.51*e* + 01	4.12**e** + 00
	Min	4.52*e* + 00	8.91*e* + 00	5.49*e* + 00	3.74*e* + 00	3.49*e* + 00	6.13*e* + 00	5.64*e* + 00	6.52*e* + 00	1.58**e** + 00

*f*_20_	*F*_mean_	3.37*e* + 02(+)	3.52*e* + 03(+)	1.05*e* + 03(+)	3.46*e* + 01(+)	3.09*e* + 03(+)	3.07*e* + 02(+)	5.33*e* + 03(+)	8.51*e* + 03(+)	5.66**e** + 00
	SD	1.51*e* + 02	8.21*e* + 03	7.76*e* + 02	8.32*e* + 00	2.51*e* + 03	1.11*e* + 02	2.01*e* + 03	5.95*e* + 03	2.37**e** + 00
	Max	7.38*e* + 02	3.36*e* + 04	4.68*e* + 03	4.79*e* + 01	1.15*e* + 04	5.43*e* + 02	9.45*e* + 03	2.86*e* + 04	1.13**e** + 01
	Min	6.94*e* + 01	2.83*e* + 02	3.23*e* + 02	6.89*e* + 00	7.50*e* + 00	1.31*e* + 02	1.16*e* + 03	1.24*e* + 03	2.25**e** + 00

*f*_21_	*F*_mean_	7.57*e* + 04(+)	1.97*e* + 05(+)	7.22*e* + 04(+)	6.57*e* + 02(+)	1.08*e* + 04(+)	1.06*e* + 03(+)	4.76*e* + 05(+)	8.06*e* + 05(+)	4.53**e** + 01
	SD	1.01*e* + 05	2.49*e* + 05	5.74*e* + 04	1.76*e* + 02	2.86*e* + 04	3.76*e* + 02	2.35*e* + 05	7.59*e* + 05	4.48**e** + 01
	Max	5.05*e* + 05	1.18*e* + 06	2.89*e* + 05	1.01*e* + 03	1.01*e* + 05	1.75*e* + 03	1.10*e* + 06	3.43*e* + 06	1.35**e** + 02
	Min	3.17*e* + 03	2.14*e* + 04	1.50*e* + 04	2.80*e* + 02	2.14*e* + 01	3.91*e* + 02	1.14*e* + 05	8.11*e* + 04	2.48**e** + 00

*f*_22_	*F*_mean_	3.53*e* + 02(+)	5.77*e* + 02(+)	2.38*e* + 02(+)	3.52*e* + 01(≈)	1.50*e* + 02(+)	2.91*e* + 02(+)	2.85*e* + 02(+)	3.69*e* + 02(+)	2.67**e** + 01
	SD	1.33*e* + 02	2.15*e* + 02	1.14*e* + 02	2.83*e* + 01	6.80*e* + 01	1.75*e* + 02	8.25*e* + 01	1.71*e* + 02	6.43**e** + 00
	Max	7.09*e* + 02	1.10*e* + 03	4.79*e* + 02	1.77*e* + 02	3.15*e* + 02	6.89*e* + 02	4.31*e* + 02	8.52*e* + 02	4.61**e** + 01
	Min	2.87*e* + 01	2.70*e* + 02	3.47*e* + 01	2.44*e* + 01	3.33*e* + 01	2.82*e* + 02	9.26*e* + 01	1.42*e* + 02	2.11**e** + 01

**Table 6:** Experimental results of PSO, PSOcf, TLBO, DE, JADE, CMA-ES, ABC, BBO, and CPDD on *f*_23_–*f*_30_ test problems of 10 variables.

*F*		PSO	PSOcf	TLBO	DE	JADE	CMA-ES	ABC	BBO	CPDD
*f*_23_	*F*_mean_	3.29*e* + 02(≈)	3.34*e* + 02(+)	3.29*e* + 02(≈)	3.29*e* + 02(≈)	3.29*e* + 02(≈)	3.29*e* + 02(≈)	2.54**e** + 02(−)	3.29*e* + 02(≈)	3.29*e* + 02
	SD	1.20*e* − 12	1.00*e* + 01	3.68*e* − 12	9.25*e* − 13	9.25*e* − 13	9.25*e* − 13	1.15*e* + 01	1.61*e* − 02	2.89**e** − 13
	Max	3.29**e** + 02	3.63*e* + 02	3.29**e** + 02	3.29**e** + 02	3.29**e** + 02	3.29**e** + 02	3.29**e** + 02	3.29*e* + 02	3.29**e** + 02
	Min	3.29*e* + 02	3.29*e* + 02	3.29*e* + 02	3.29*e* + 02	3.29*e* + 02	3.29*e* + 02	1.10**e** + 01	3.29*e* + 02	3.29*e* + 02

*f*_24_	*F*_mean_	1.21*e* + 02(+)	1.23*e* + 02(+)	1.10*e* + 02(+)	1.29*e* + 02(+)	1.08*e* + 02(≈)	2.04*e* + 02(+)	1.17*e* + 02(+)	1.25*e* + 02(+)	1.07**e** + 02
	SD	5.31*e* + 00	1.07*e* + 01	4.57*e* + 00	3.12*e* + 00	2.15*e* + 00	1.58*e* + 02	1.93*e* + 01	6.82*e* + 00	2.43**e** + 00
	Max	1.34*e* + 02	1.58*e* + 02	1.22*e* + 02	1.33*e* + 02	1.12*e* + 02	1.00*e* + 03	1.36*e* + 02	1.40*e* + 02	1.09**e** + 02
	Min	1.11*e* + 02	1.10*e* + 02	1.00**e** + 02	1.20*e* + 02	1.00**e** + 02	1.15*e* + 02	1.90*e* + 01	1.13*e* + 02	1.00**e** + 02

*f*_25_	*F*_mean_	1.83*e* + 02(+)	1.97*e* + 02(+)	1.25*e* + 02(+)	1.62*e* + 02(+)	1.39*e* + 02(+)	1.97*e* + 02(+)	1.38*e* + 02(+)	1.64*e* + 02(+)	1.15**e** + 02
	SD	3.29*e* + 01	1.69*e* + 01	1.58*e* + 01	3.96*e* + 01	3.89*e* + 01	1.44*e* + 01	8.60*e* + 00	3.41*e* + 01	7.17**e** + 00
	Max	2.02*e* + 02	2.04*e* + 02	1.77*e* + 02	2.01*e* + 02	2.01*e* + 02	2.04*e* + 02	1.60*e* + 02	2.03*e* + 02	1.23**e** + 02
	Min	1.21*e* + 02	1.34*e* + 02	1.00**e** + 02	1.00**e** + 02	1.00**e** + 02	1.35*e* + 02	1.22*e* + 02	1.15*e* + 02	1.00**e** + 02

*f*_26_	*F*_mean_	1.00**e** + 02(≈)	1.00**e** + 02(≈)	1.00**e** + 02(≈)	1.00**e** + 02(≈)	1.00**e** + 02(≈)	2.14*e* + 02(+)	1.00**e** + 02(≈)	1.00*e* + 02(≈)	1.00**e** + 02
	SD	4.73*e* − 02	1.65*e* − 01	3.15*e* − 02	2.65*e* − 02	1.57*e* − 02	1.22**e** − 02	1.03*e* + 00	7.02*e* − 02	2.28*e* − 02
	Max	1.00**e** + 02	1.00**e** + 02	1.00**e** + 02	1.00**e** + 02	1.00**e** + 02	5.75*e* + 02	1.00**e** + 02	1.00*e* + 02	1.00**e** + 02
	Min	1.00*e* + 02	1.00*e* + 02	1.00*e* + 02	1.00*e* + 02	1.00*e* + 02	8.84**e** + 01	9.45*e* + 01	1.00*e* + 02	1.00*e* + 02

*f*_27_	*F*_mean_	3.03*e* + 02(+)	2.92*e* + 02(+)	1.81*e* + 02(+)	8.82*e* + 01(+)	4.92*e* + 01(+)	3.37*e* + 02(+)	8.06*e* + 01(+)	3.19*e* + 02(+)	1.23**e** + 00
	SD	1.06*e* + 02	1.94*e* + 02	1.68*e* + 02	1.60*e* + 02	1.23*e* + 02	9.90*e* + 01	1.42*e* + 02	1.45*e* + 02	3.23**e** − 01
	Max	4.04*e* + 02	4.89*e* + 02	4.00*e* + 02	4.00*e* + 02	4.00*e* + 02	4.00*e* + 02	4.00*e* + 02	4.49*e* + 02	2.09**e** + 00
	Min	2.46*e* + 00	1.55*e* + 00	2.54*e* + 00	1.53*e* + 00	1.16*e* + 00	4.03*e* + 00	5.01*e* + 00	3.92*e* + 00	7.26**e** − 01

*f*_28_	*F*_mean_	4.70*e* + 02(+)	4.70*e* + 02(+)	3.93*e* + 02(+)	3.80*e* + 02(≈)	4.01*e* + 02(+)	1.58*e* + 03(+)	3.58**e** + 02(≈)	4.15*e* + 02(+)	3.71*e* + 02
	SD	7.30*e* + 01	8.13*e* + 01	3.18*e* + 01	3.78*e* + 01	4.83*e* + 01	1.26*e* + 03	5.81*e* + 01	4.78*e* + 01	1.36**e** + 00
	Max	6.75*e* + 02	6.65*e* + 02	4.80*e* + 02	4.82*e* + 02	4.78*e* + 02	4.58*e* + 03	3.83*e* + 02	5.20*e* + 02	3.72**e** + 02
	Min	3.71*e* + 02	3.92*e* + 02	3.69*e* + 02	3.56*e* + 02	3.56*e* + 02	3.72*e* + 02	1.04**e** + 02	3.58*e* + 02	3.69*e* + 02

*f*_29_	*F*_mean_	4.28*e* + 05(+)	1.26*e* + 05	5.93*e* + 02(+)	2.18**e** + 02(≈)	2.55*e* + 02(+)	5.77*e* + 04(+)	3.45*e* + 02(+)	4.73*e* + 02(+)	2.22*e* + 02
	SD	1.00*e* + 06	9.79*e* + 04	1.36*e* + 02	1.54*e* + 01	4.53*e* + 01	1.14*e* + 05	4.71*e* + 01	2.59*e* + 02	7.94**e** − 01
	Max	3.62*e* + 06	2.04*e* + 06	9.05*e* + 02	2.23**e** + 02	3.54*e* + 02	1.72*e* + 06	4.53*e* + 02	1.63*e* + 03	2.25*e* + 02
	Min	3.36*e* + 02	3.04*e* + 02	3.08*e* + 02	1.37**e** + 02	2.21*e* + 02	1.44*e* + 02	2.44*e* + 02	2.63*e* + 02	2.21*e* + 02

*f*_30_	*F*_mean_	8.09*e* + 02(+)	8.51*e* + 02(+)	5.44*e* + 02(+)	4.62**e** + 02(≈)	4.91*e* + 02(+)	1.05*e* + 03(+)	6.32*e* + 02(+)	7.75*e* + 02(+)	4.62**e** + 02
	SD	2.62*e* + 02	3.91*e* + 02	1.03*e* + 02	3.64*e* + 00	3.04*e* + 01	3.62*e* + 02	1.16*e* + 02	1.81*e* + 02	1.49**e** + 00
	Max	1.52*e* + 03	2.46*e* + 03	9.95*e* + 02	4.80*e* + 02	5.80*e* + 02	1.88*e* + 03	1.01*e* + 03	1.22*e* + 03	4.62**e** + 02
	Min	5.09*e* + 02	4.67*e* + 02	4.59*e* + 02	4.54**e** + 02	4.62*e* + 02	5.50*e* + 02	5.00*e* + 02	5.35*e* + 02	4.54**e** + 02

**Table 7:** Experimental results of PSO, PSOcf, TLBO, DE, JADE, CMA-ES, ABC, BBO, and CPDD on *f*_23_–*f*_30_ test problems of 30 variables.

*F*		PSO	PSOcf	TLBO	DE	JADE	CMA-ES	ABC	BBO	CPDD
*f*_23_	*F*_mean_	3.15**e** + 02(≈)	3.51*e* + 02(+)	3.15**e** + 02(≈)	3.15**e** + 02(≈)	3.15**e** + 02(≈)	3.15**e** + 02(≈)	3.15**e** + 02(≈)	3.16*e* + 02(+)	3.15**e** + 02
	SD	2.21*e* − 01	3.01*e* + 01	1.78*e* − 12	1.38*e* − 12	1.37**e** − 12	5.20*e* − 12	7.91*e* − 02	5.95*e* − 01	1.38*e* − 12
	Max	3.16*e* + 02	4.61*e* + 02	3.15**e** + 02	3.15**e** + 02	3.15**e** + 02	3.15**e** + 02	3.15**e** + 02	3.17*e* + 02	3.15**e** + 02
	Min	3.15**e** + 02	3.15**e** + 02	3.15**e** + 02	3.15**e** + 02	3.15**e** + 02	3.15**e** + 02	3.15**e** + 02	3.15*e* + 02	3.15**e** + 02

*f*_24_	*F*_mean_	2.34*e* + 02(+)	2.53*e* + 02(+)	2.00**e** + 02(−)	2.16*e* + 02(+)	2.24*e* + 02(+)	2.62*e* + 02(+)	2.27*e* + 02(+)	2.32*e* + 02(+)	2.06*e* + 02
	SD	8.37*e* + 00	1.56*e* + 01	1.06**e** − 03	1.03*e* + 01	1.09*e* + 00	1.32*e* + 02	6.41*e* − 01	3.73*e* + 00	1.00*e* + 01
	Max	2.50*e* + 02	2.79*e* + 02	2.00**e** + 02	2.27*e* + 02	2.28*e* + 02	9.06*e* + 02	2.28*e* + 02	2.48*e* + 02	2.21*e* + 02
	Min	2.23*e* + 02	2.24*e* + 02	2.00**e** + 02	2.00**e** + 02	2.22*e* + 02	2.23*e* + 02	2.25*e* + 02	2.27*e* + 02	2.00**e** + 02

*f*_25_	*F*_mean_	2.09*e* + 02(−)	2.13*e* + 02(−)	2.00**e** + 02(−)	2.02*e* + 02(−)	2.04*e* + 02(−)	2.04*e* + 02(−)	2.08*e* + 02(−)	2.07*e* + 02(−)	2.18*e* + 02
	SD	2.81*e* + 00	8.57*e* + 00	1.55*e* + 00	9.80*e* − 02	1.64*e* + 00	2.38*e* + 00	1.11**e** + 00	1.61*e* + 00	8.25*e* + 00
	Max	2.17*e* + 02	2.34*e* + 02	2.06**e** + 02	2.03*e* + 02	2.09*e* + 02	2.12*e* + 02	2.09*e* + 02	2.11*e* + 02	2.23*e* + 02
	Min	2.05*e* + 02	2.03*e* + 02	2.00*e* + 02	2.02*e* + 02	2.02*e* + 02	2.02*e* + 02	2.05*e* + 02	2.04*e* + 02	2.00**e** + 02

*f*_26_	*F*_mean_	1.27*e* + 02(+)	1.19*e* + 02(+)	1.00**e** + 02(≈)	1.00**e** + 02(≈)	1.00**e** + 02(≈)	1.14*e* + 02(≈)	1.00**e** + 02(≈)	1.00**e** + 02(≈)	1.00**e** + 02
	SD	4.49*e* + 01	3.70*e* + 01	1.06*e* − 01	3.37*e* − 02	3.24**e** − 02	5.43*e* + 01	5.63*e* − 02	1.20*e* − 01	5.57*e* − 02
	Max	2.01*e* + 02	2.01*e* + 02	1.00**e** + 02	1.00**e** + 02	1.00**e** + 02	3.23*e* + 02	1.00**e** + 02	1.00**e** + 02	1.00**e** + 02
	Min	1.00**e** + 02	1.00**e** + 02	1.00**e** + 02	1.00**e** + 02	1.00**e** + 02	1.00**e** + 02	1.00**e** + 02	1.00**e** + 02	1.00**e** + 02

*f*_27_	*F*_mean_	6.49*e* + 02(+)	8.62*e* + 02(+)	5.24*e* + 02(+)	3.45*e* + 02(+)	3.34*e* + 02(+)	3.97*e* + 02(+)	4.09*e* + 02(+)	6.07*e* + 02(+)	3.00**e** + 02
	SD	1.27*e* + 02	2.40*e* + 02	1.27*e* + 02	4.84*e* + 01	4.76*e* + 01	9.75*e* + 01	2.74*e* + 00	1.37*e* + 02	1.19**e** − 13
	Max	8.55*e* + 02	1.17*e* + 03	7.51*e* + 02	4.00*e* + 02	4.12*e* + 02	7.95*e* + 02	4.14*e* + 02	8.38*e* + 02	3.00**e** + 02
	Min	4.05*e* + 02	4.13*e* + 02	4.01*e* + 02	3.00**e** + 02	3.00**e** + 02	3.01*e* + 02	4.04*e* + 02	4.02*e* + 02	3.00**e** + 02

*f*_28_	*F*_mean_	1.31*e* + 03(+)	1.70*e* + 03(+)	1.02*e* + 03(+)	7.93*e* + 02(−)	7.83**e** + 02(−)	4.42*e* + 03(+)	9.63*e* + 02(+)	9.25*e* + 02(+)	8.40*e* + 02
	SD	3.73*e* + 02	4.47*e* + 02	1.34*e* + 02	4.53*e* + 01	4.69*e* + 01	2.85*e* + 03	4.52*e* + 01	1.14*e* + 02	2.06**e** + 01
	Max	2.37*e* + 03	2.99*e* + 03	1.38*e* + 03	8.45*e* + 02	8.33**e** + 02	9.37*e* + 03	1.09*e* + 03	1.47*e* + 03	8.82*e* + 02
	Min	9.29*e* + 02	1.04*e* + 03	8.16*e* + 02	6.79**e** + 02	6.80*e* + 02	7.09*e* + 02	8.60*e* + 02	7.87*e* + 02	8.00*e* + 02

*f*_29_	*F*_mean_	9.06*e* + 05(+)	6.34*e* + 06(+)	1.45*e* + 06(+)	7.20**e** + 02(≈)	7.88*e* + 02(≈)	7.89*e* + 02(+)	1.08*e* + 03(+)	1.84*e* + 03(+)	7.30*e* + 02
	SD	1.95*e* + 06	6.24*e* + 06	3.30*e* + 06	1.88**e** + 01	2.26*e* + 02	7.02*e* + 01	6.67*e* + 01	4.51*e* + 02	2.56*e* + 01
	Max	2.71*e* + 07	2.68*e* + 07	9.18*e* + 06	8.18**e** + 02	1.64*e* + 03	1.00*e* + 03	1.20*e* + 03	2.95*e* + 03	8.53*e* + 02
	Min	5.34**e** + 02	3.96*e* + 04	8.84*e* + 02	7.14*e* + 02	7.15*e* + 02	5.81*e* + 02	8.80*e* + 02	1.26*e* + 03	7.14*e* + 02

*f*_30_	*F*_mean_	6.59*e* + 03(+)	1.19*e* + 05(+)	3.14*e* + 03(+)	5.82**e** + 02(−)	1.84*e* + 03(+)	2.16*e* + 03(+)	4.08*e* + 03(+)	6.09*e* + 03(+)	9.74*e* + 02
	SD	9.18*e* + 03	8.99*e* + 04	1.64*e* + 03	2.23*e* + 02	7.33*e* + 02	5.14*e* + 02	7.43*e* + 02	2.61*e* + 03	2.12**e** + 02
	Max	5.23*e* + 04	3.29*e* + 05	7.00*e* + 03	1.37*e* + 03	3.74*e* + 03	3.14*e* + 03	5.77*e* + 03	1.13*e* + 04	1.30**e** + 03
	Min	2.47*e* + 03	4.96*e* + 03	7.81*e* + 03	3.96**e** + 02	8.25*e* + 02	1.22*e* + 03	2.21*e* + 03	2.31*e* + 03	4.94*e* + 02

**Table 8:** Comparison of CPDD with PSO, PSOcf, TLBO, DE, JADE, CMA-ES, ABC, and BBO on the CEC2014 benchmarks (*D* = 10 and 30 dimensions).

*D*		PSO	PSOcf	TLBO	DE	JADE	CMA-ES	ABC	BBO
10	+	26	26	27	20	18	25	23	25
	−	1	1	1	1	3	0	3	3
	≈	3	3	2	9	9	5	4	2

30	+	28	26	26	20	14	22	21	22
	−	1	2	2	3	9	4	6	6
	≈	1	2	2	7	7	4	3	2

**Table 9:** Comparisons between PSO, PSOcf, TLBO, DE, JADE, CMA-ES, ABC, BBO, and CPDD on MSE.

Algorithm	Training error		Testing error	
	Mean	Std.	Mean	Std.
PSO	4.52*e* − 04	8.25*e* − 04	1.54*e* − 02	5.44*e* − 02
PSOcf	3.38*e* − 04	7.77*e* − 04	4.52*e* − 04	1.19*e* − 03
TLBO	3.41*e* − 03	2.45*e* − 03	2.68*e* − 03	2.22*e* − 03
DE	3.31*e* − 03	1.59*e* − 03	4.31*e* − 03	2.31*e* − 03
JADE	1.15*e* − 03	7.90*e* − 04	1.07*e* + 00	2.78*e* + 00
CMA-ES	8.65*e* − 03	1.49*e* − 02	5.05*e* − 03	9.27*e* − 03
ABC	1.32*e* − 03	1.32*e* − 03	1.53*e* − 02	4.03*e* − 02
BBO	3.13*e* − 03	7.46*e* − 03	8.65*e* − 02	2.47*e* − 01
CPDD	7.31*e* − 05	9.01*e* − 05	3.70*e* − 04	8.54*e* − 04

**Table 10:** Comparisons between PSO, PSOcf, TLBO, DE, JADE, CMA-ES, ABC, BBO, and CPDD on MSE.

Algorithm	Training error		Testing error	
	Mean	Std.	Mean	Std.
PSO	4.83*e* − 04	3.36*e* − 04	5.02*e* − 04	3.50*e* − 04
PSOcf	1.11*e* − 03	9.93*e* − 04	1.16*e* − 03	1.05*e* − 03
TLBO	4.88*e* − 03	2.32*e* − 03	5.00*e* − 03	2.45*e* − 03
DE	4.54*e* − 03	3.71*e* − 03	4.69*e* − 03	3.80*e* − 03
JADE	2.00*e* − 03	8.51*e* − 04	6.78*e* − 01	2.87*e* + 00
CMA-ES	3.14*e* − 02	3.83*e* − 02	3.17*e* − 02	3.72*e* − 02
ABC	3.61*e* − 03	1.76*e* − 03	3.76*e* − 03	1.87*e* − 03
BBO	1.50*e* − 03	1.19*e* − 03	1.57*e* − 03	1.26*e* − 03
CPDD	2.90*e* − 04	2.44*e* − 04	3.00*e* − 04	2.55*e* − 04
